# Estimates of bilinear pseudodifferential operators associated to bilinear Hörmander classes in Besov and Triebel–Lizorkin spaces with variable exponents

**DOI:** 10.1186/s13660-018-1759-y

**Published:** 2018-07-11

**Authors:** Jingshi Xu, Jinlai Zhu

**Affiliations:** School of Mathematics and Statistics, Hainan Normal University, Haikou, People’s Republic of China

**Keywords:** 47G30, 46E35, 42B25, 42B35, Variable exponent, Triebel–Lizorkin space, Besov space, Bilinear pseudodifferential operator

## Abstract

In this paper, we give Leibniz-type estimates of bilinear pseudodifferential operators associated to bilinear Hörmander classes in Besov and Triebel–Lizorkin spaces with variable exponents. To obtain the estimate for Triebel–Lizorkin spaces with variable exponents, we present their approximation characterization.

## Introduction

The theory of bilinear pseudodifferential operators with symbols in the Hörmander classes has been extensively studied by many authors. Different from their linear counterparts $\mathcal{S}_{\rho,\delta}^{0}$, $0\leq\delta\leq\rho<1$, whose corresponding pseudodifferential operators are bounded on ${L}^{2} ( \mathbb{{R}}^{{n}} )$, the classes ${B} \mathcal{S}_{\rho,\delta}^{0}$ (its definition is in Sect. 2) contain symbols for which the corresponding bilinear pseudodifferential operators do not map any product ${L}^{{P}_{1}} ( \mathbb{{R}}^{{n}} ) \times {L}^{{P}_{2}} ( \mathbb{{R}}^{{n}} )$, into any ${L}^{{P}} ( \mathbb{{R}}^{{n}} )$ with ${1} / {{P}} = {1} / {{P}_{1}} + {1} / {{P}_{2}}$; see [6]. Moreover, ${B} \mathcal{S}_{1,1}^{0}$ contains symbols for which the corresponding bilinear operators are unbounded from any ${L}^{{P}_{1}} ( \mathbb{{R}}^{{n}} ) \times{L}^{{P}_{2}} ( \mathbb{{R}}^{{n}} )$ into any ${L}^{{P}} ( \mathbb{{R}}^{{n}} )$ with ${1} / {{P}} = {1} / {{P}_{1}} + {1} / {{P}_{2}}$. Nevertheless, the operators with symbols in ${B} \mathcal{S}_{1,1}^{0}$ are proved to be bounded on products of Sobolev spaces with positive smoothness in [8]. However, the classes ${B} \mathcal{S}_{\rho,\delta}^{0}$ with $0\leq\delta<1$, like their linear setting, the corresponding bilinear pseudodiffer ential operators are bilinear Calderón–Zygmund operators. In [7], the properties of symbols, and boundedness properties of bilinear pseudodifferential operators in Lebesgue spaces were given. For pseudodifferential operators with symbols in the bilinear Hörmander classes of sufficiently negative order, their boundedness properties in Lebesgue spaces, weak-type spaces, BMO and Sobolev spaces are established in [6]. In [8], by establishing a symbolic calculus for the transposes of a class of bilinear pseudodifferential operators, Benyi and Torres proved that these operators are bounded on products of Lebesgue spaces. In [24], Herbert and Naibo showed that bilinear pseudodifferential operators with symbols in Besov spaces are bounded on products of Lebesgue spaces. In [36], Miyachi and Tomita determined the order m for which all the bilinear pseudodifferential operators with symbols in the Hörmander class ${B} \mathcal{S}_{0,0}^{{m}}$ are bounded among Lebesgue spaces, local Hardy spaces, and bmo spaces. In [35], Michalowski, Rule and Staubach obtained the boundedness of multilinear pseudodifferential operators with symbols which are only measurable in the spatial variables in Lebesgue spaces and the boundedness of bilinear pseudodifferential operators with symbols in the Hörmander classe ${B} \mathcal{S}_{\rho,\delta}^{{m}}$. In [42], Rodríguez-López and Staubach obtained the boundedness of rough Fourier integral and pseudodifferential operators. As applications, then they considered boundedness results for Hörmander class bilinear pseudodifferential operators, certain classes of bilinear (as well as multilinear) Fourier integral operators, and rough multilinear operators. Recently, in [37] Naibo obtained boundedness properties on the scales of inhomogeneous Triebel–Lizorkin and Besov spaces of positive smoothness for pseudodifferential operators with symbols in certain bilinear Hörmander classes.

Since variable exponent function spaces have widely used in many fields such as electrorheological fluid [43], differential equations [19, 23, 41] and image restoration [9, 22, 29, 34, 46], many classical constant exponent function spaces have been generalized to variable exponent setting, such as variable exponent Bessel potential spaces [4, 21], variable Hajłasz–Sobolev spaces [5], variable exponent Besov and Triebel–Lizorkin spaces [3, 13, 16, 27, 30, 31, 49], variable exponent Hardy spaces [38, 56], variable exponent Morrey spaces [2], variable exponent Herz spaces [1, 26, 44], variable exponent Herz-type Hardy spaces [18, 28, 48], variable exponent Herz–Morrey Hardy spaces [50], variable exponent Herz-type Besov and Triebel–Lizorkin spaces [14, 17, 45, 52], variable exponent Morrey-type Besov and Triebel–Lizorkin spaces [20], Herz–Morrey-type Besov and Triebel–Lizorkin spaces with variable exponents [15], Triebel–Lizorkin-type spaces with variable exponents [57], variable weak Hardy spaces [53], Besov-type spaces with variable smoothness and integrability [58], variable integral and smooth exponent Triebel–Lizorkin spaces associated with a non-negative self-adjoint operator [51], variable exponent Hardy spaces associated with operators [55], and variable Hardy spaces associated with operators [54, 59, 60]. For the boundedness of integral operators in variable function spaces, we recommend [32] and [33]. In [39], Noi gave Fourier multiplier theorems for Besov and Triebel–Lizorkin spaces with variable exponents. Motivated by the mentioned work, we shall present the boundedness of the bilinear pseudodifferential operator associated to bilinear Hörmander classes in Besov and Triebel–Lizorkin spaces with variable exponents. Indeed, by using the embedding properties of the Besov and Triebel–Lizorkin spaces with variable exponents, we shall establish corresponding Leibnitz-type inequalities for the Besov and Triebel–Lizorkin spaces with variable exponents.

The plan of the paper is as follows. In Sect. 2, we shall state notions, preliminary results. In particular, we give the approximation characterizations of Triebel–Lizorkin spaces with variable exponents. In Sect. 3, we present the proofs of the main results.

## Preliminaries

We denote by $\mathcal{S} ( \mathbb{{R}}^{{n}} )$ the usual Schwartz space of rapidly decreasing complex-valued functions and $\mathcal{S}' ( \mathbb{{R}}^{{n}} )$ the dual space of tempered distributions. As usual, we denote by *f̂* or $\mathcal{F} ({f})$ the Fourier transform of $f\in\mathcal{S}' ( \mathbb{{R}}^{{n}} )$. In particular, we use the formula

$$\widehat{{f}} ( \xi ): = \int_{\mathbb{R}^{n}} f ( x ) e^{ - 2\pi ix\xi} \,{dx}\quad \mbox{if } f \in L^{1} \bigl( \mathbb{R}^{n} \bigr). $$

We denote by $\mathcal{F}^{-1} ({f})$ or *f̌* the inverse Fourier transform of *f*. Given a real number ${r} \geq0$, the homogeneous derivative of order *r*, ${D}^{{r}}$, is defined by

$$\widehat{{D}^{{r}}{f}} ( \xi ): = \vert \xi \vert ^{{r}} \widehat{ {f}} ( \xi ),\quad\xi\in\mathbb{R}^{n}. $$

Let ${m} \in\mathbb{R}$ and $0\leq\delta\leq\rho\leq1$. A function *σ* on $\mathbb{{R}}^{3{n}}$, is an element of the bilinear Hörmander class ${B} \mathcal{S}_{\rho,\delta}^{{m}}$ if for all multi-indices $\gamma ,\alpha,\beta\in \mathbb{{N}}_{0}^{{n}}$ there exist some positive constants $C_{\gamma ,\alpha,\beta}$ such that

$$\bigl\vert \partial_{x}^{\gamma} \partial_{\xi}^{\alpha} \partial_{\eta}^{\beta} \sigma ( x,\xi,\eta ) \bigr\vert \le C_{\gamma,\alpha,\beta} \bigl( 1 + \vert \xi \vert + \vert \eta \vert \bigr)^{m + \delta \vert \gamma \vert - \rho ( \vert \alpha \vert + \vert \beta \vert )} $$

for all ${x},\xi,\eta\in\mathbb{{R}}^{{n}}$, where $\vert \gamma \vert $ denotes the sum of its components, $\vert \alpha \vert $ and $\vert \beta \vert $ are similar. The bilinear pseudodifferential operator associated to *σ* is defined by

$$T_{\sigma} ( f,g ) ( x ): = \int_{\mathbb{R}^{2n}} \sigma ( x,\xi,\eta ) \widehat{ f} ( \xi )\widehat{ g} ( \eta )e^{2\pi ix ( \xi+ \eta )}\,{d}\xi \,{d}\eta,\quad x \in\mathbb{R}^{n},f,g \in\mathcal {{S}} \bigl(\mathbb{R}^{n} \bigr). $$

Let $\sigma\in{B} \mathcal{S}_{\rho,\delta}^{{m}}$ and ${N},{M}\in\mathbb{{N}}_{0}:= \mathbb{N} \cup \{ 0 \}$. Define

$$\Vert \sigma \Vert _{N,M}: = \sup_{ \vert \gamma \vert \le N, \vert \alpha \vert + \vert \beta \vert \le M}\sup _{x,\xi,\eta\in \mathbb{R}^{n}} \bigl\vert \partial_{x}^{\gamma} \partial_{\xi}^{\alpha} \partial_{\eta}^{\beta} \sigma_{j,k,\ell} ( x,\xi,\eta ) \bigr\vert \bigl( 1 + \vert \xi \vert + \vert \eta \vert \bigr)^{ - m - \delta \vert \gamma \vert + \rho ( \vert \alpha \vert + \vert \beta \vert )}. $$

Then ${B} \mathcal{S}_{\rho,\delta}^{{m}}$ becomes a Fréchet space with the family of norms $\{ \Vert \sigma \Vert _{{N},{M}}: {N},{M}\in\mathbb{{N}}_{0} \}$.

If ${a}\leq{cb}$ and $b\leq{ca}$ we will write ${a}\approx {b}$. *C* is always a positive constant but it may change from line to line.

For a measurable function *p* on $\mathbb{{R}}^{{n}}$, we denote ${p}^{-} :=\operatorname{ess} \inf_{{x} \in \mathbb{{R}}^{{n}}} {p}({x})$ and ${p}^{+} := \operatorname{ess} \sup_{{x} \in \mathbb{{R}}^{{n}}} {p}({x})$. We denote by $\mathcal{P}_{0}$ the subset of measurable functions on $\mathbb{{R}}^{{n}}$ with values in $(0,\infty]$ such that ${p}^{-} > 0$, and by $\mathcal{P}$ the subset of measure functions with values in $[1,\infty]$. For ${p} ( {\cdot} ) \in \mathcal{P}_{0}$, the function $\rho_{{p}}$ is defined as follows:

$$\rho_{p}(t): = \left \{ \textstyle\begin{array}{l@{\quad}l} {t}^{p} & \mbox{if } p \in(0,\infty),\\ 0 & \mbox{if } p = \infty \mbox{ and } t \le1, \\ \infty& \mbox{if } p = \infty \mbox{ and } t \ge1. \end{array}\displaystyle \right . $$

The convention $1^{\infty} =0$ is adopted in order for $\rho_{{p}}$ to be left-continuous. The variable exponent modular is defined by

$$\rho_{p ( \cdot )} ( f ): = \int_{\mathbb{R}^{n}} \rho_{p ( x )} \bigl( \bigl\vert f ( x ) \bigr\vert \bigr)\,{dx}. $$

The variable exponent Lebesgue space ${L}^{{p}({\cdot})}$ consists of measurable functions $f: \mathbb{{R}}^{{n}}\rightarrow \mathbb{{R}}$, with $\rho_{{p} ( {\cdot} )} ( \lambda{f} ) < \infty$ for some $\lambda>0$. The Luxemburg (quasi)-norm on this space is defined by the formula

$$\Vert f \Vert _{p( \cdot)}: = \inf \bigl\{ \lambda> 0: \rho_{p( \cdot )} ( f / \lambda ) \le1 \bigr\} . $$

Let ${p},{q}\in \mathcal{P}_{0}$. For a sequence of ${L}^{{p}({\cdot})}$-functions $( {f}_{{v}} )_{{v}}$, we define the modular

$$\rho_{\ell^{q( \cdot)}(L^{p( \cdot)})} \bigl( ( f_{\nu} )_{\nu} \bigr): = \sum _{{\nu}} \bigl\{ \inf\lambda_{\nu } > 0: \rho_{p( \cdot)} \bigl( f_{\nu} / \lambda_{{\nu}}^{\frac{1}{q( \cdot)}} \bigr) \le1 \bigr\} , $$

where we use the convention $\lambda^{\frac{1}{\infty}} =1$. Then the norm in the mixed Lebesgue-sequence space $\ell^{{q}({\cdot})} ( {L}^{{p}({\cdot})} )$ is defined by

$$\bigl\Vert ( f_{\nu} )_{\nu} \bigr\Vert _{\ell^{q( \cdot)} ( L^{p( \cdot)} )}: = \inf \biggl\{ \mu> 0:\rho_{\ell^{q( \cdot )} ( L^{p( \cdot)} )} \biggl( \frac{1}{\mu} ( f_{\nu} )_{\nu} \biggr) \le1 \biggr\} . $$

If ${q}^{+} < \infty$, then

$$\inf \bigl\{ \lambda> 0:\rho_{p( \cdot)} \bigl( f / \lambda^{\frac{1}{q( \cdot)}} \bigr) \le1 \bigr\} = \| | f |^{{q( \cdot)}} \|_{\frac{p( \cdot)}{^{q( \cdot)}}}. $$

Since the above right-hand side expression is much simpler, we use this notation to represent the above left-hand side even when ${q}^{+} =\infty$, and that means

$$\rho_{\ell^{q( \cdot)} ( L^{p( \cdot)} )} \bigl( ( f_{\nu} )_{\nu} \bigr) = \sum _{v} \| | f_{v} |^{{q( \cdot )}} \|_{\frac{p( \cdot)}{^{q( \cdot)}}} $$

for the modular.

Let $( {f}_{{v}} )_{{v}\in \mathbb{{N}}_{0}}$ be a sequence of measurable functions on $\mathbb{{R}}^{{n}}$, then the norm of $( {f}_{{v}} )_{{v}\in \mathbb{{N}}_{0}}$ in the space ${L}^{{p}({\cdot})} ( \ell^{{q}({\cdot})} )$ is defined by

$$\bigl\Vert ( f_{\nu} )_{\nu} \bigr\Vert _{L^{p( \cdot)} ( \ell^{q( \cdot)} )}: = \Biggl\Vert \Biggl( \sum_{\nu= 0}^{\infty} \bigl\vert f_{\nu} ( \cdot) \bigr\vert ^{{q( \cdot)}} \Biggr)^{\frac{1}{^{q( \cdot)}}} \Biggr\Vert _{L^{p( \cdot)}}. $$

In the development of the variable exponent function spaces, the concept of log-Hölder continuity is the cornerstone, which was introduced in [10, 11].

### Definition 2.1

Let g be a real function on $\mathbb{{R}}^{{n}}$.

(i) The function g is called locally log-Hölder continuous, abbreviated ${g}\in{C}_{{\mathrm{loc}}}^{\log} $, if there exists ${C}_{\log} > 0$ such that
  $$\bigl\vert g(x) - g(y) \bigr\vert \le\frac{C_{\log}}{\log ( e + 1 / \vert {x} - {y} \vert )}, \quad x,y \in \mathbb{R}^{n}, \vert x - y \vert < \frac{1}{2}. $$
(ii) The function g is called globally log-Hölder continuous, abbreviated ${g}\in{C}_{\log} $, if it is locally log-Hölder continuous and there exists ${g}_{\infty} \in\mathbb{{R}}$ such that
  $$\bigl\vert g(x) - g_{\infty} \bigr\vert \le\frac{C_{\log}}{\log ( e + \vert {x} \vert )}, \quad \forall x \in\mathbb{R}^{n}. $$

The notation $\mathcal{P}^{\log}$ is used for those variable exponents ${p}\in \mathcal{P}$ with $\frac{1}{{p}} \in{C}_{\log} $. The class $\mathcal{P}_{0}^{\log}$ is defined analogously. Let *f*, *g* be in ${L}^{1} ( \mathbb {{R}}^{{n}} )$. Define the convolution ${f}*{g} $ by

$$( f * g ) ( x ) = \int_{\mathbb{R}^{n}} f ( x - y )g ( y )\,{d}y. $$

If ${p}\in\mathcal{P}^{\log} $, then convolution with a radially decreasing ${L}^{1}$-function is bounded on ${L}^{{P}({\cdot})}$: $\Vert \varphi *f \Vert _{{p}({\cdot})} \leq{c} \Vert \varphi \Vert _{1} \Vert {f} \Vert _{{p}({\cdot})} $.

### Definition 2.2

Let *ψ* be a function in $\mathcal{S} ( \mathbb{{R}}^{{n}} )$ satisfying $\psi ( {x} ) =1$ for $| {x}|\leq1$ and $\psi ( {x} ) =0$ for $| {x}|\geq2$. We let $\hat{\varphi}_{0} ( {x} ) := \psi ( {x} )$, $\hat{\varphi} ( 2{x} ) := \psi ( {x} ) - \psi ( 2{x} )$ and $\varphi_{{j}} ( {x} ) := 2^{{jn}} \varphi( 2^{{j}} {x})$ for ${j}\in\mathbb{{N}}$ and for all ${x}\in \mathbb{{R}}^{{n}}$. Then $\sum_{{k}\in\mathbb{{N}}_{0}} \hat{\varphi}_{{k}} =1$.

Thus we obtain the Littlewood–Paley decomposition ${f}= \sum_{{v}=0}^{\infty} \varphi_{{v}} *{f}$ for all ${f}\in \mathcal{S}' ( \mathbb{{R}}^{{n}} )$ (convergence in $\mathcal{S}' ( \mathbb{{R}}^{{n}} )$).

We also put $\hat{\psi}_{0} := \hat{\varphi}_{0} + \hat{\varphi}_{1}$ and $\hat{\psi}_{k} := \hat{\varphi}_{{k}-1} + \hat{\varphi}_{{k}} + \hat{\varphi}_{{k}+1}$ for ${k}\in \mathbb{{N}}$. It is easy to see that $\hat{\varphi}_{{k}} \hat{\psi}_{k} = \hat{\varphi}_{{k}}$ for ${k}\in\mathbb{{N}}_{0}$ and

$$\begin{gathered}\operatorname{supp} ( \widehat{\psi}_{{k}} ) \subset \bigl\{ \xi\in\mathbb{R}^{n}:2^{k - 2} \le \vert \xi \vert \le2^{k + 2} \bigr\} \quad\mbox{for } k \ge2, \\ \operatorname{supp} ( \widehat{\psi}_{{k}} ) \subset \bigl\{ \xi\in \mathbb{R}^{n}: \vert \xi \vert \le2^{k + 2} \bigr\} \quad \mbox{for } k = 0,1. \end{gathered} $$

For an appropriate function *h*, $h(D)$ will stand for the multiplier operator given $\hat{{h}({D}){f}} ={h} \hat{{f}}$ for ${f}\in\mathcal{S}' ( \mathbb{{R}}^{{n}} )$.

### Definition 2.3

Let $\varphi_{{v}}$ be as in Definition 2.2. For ${s}: \mathbb{{R}}^{{n}}\rightarrow\mathbb{{R}}$ and ${p},{q} \in\mathcal{P}_{0}$.

(i) Let ${p},{q}\in \mathcal{P}_{0}^{\log} ( \mathbb{{R}}^{{n}} )$ and let ${s} \in {C}_{{\mathrm{loc}}}^{\log} ( \mathbb{{R}}^{{n}} )$. Then
  $$F_{p( \cdot),q( \cdot)}^{s( \cdot)} \bigl(\mathbb{R}^{n} \bigr): = \bigl\{ f \in\mathcal {{S}}' \bigl(\mathbb{R}^{n} \bigr): \Vert f \Vert _{F_{p( \cdot),q( \cdot )}^{s( \cdot)}}^{\varphi} < \infty \bigr\} , $$
  where
  $$\Vert f \Vert _{F_{p( \cdot),q( \cdot)}^{s( \cdot)}}^{\varphi}: = \bigl\Vert \bigl( 2^{js( \cdot)}\varphi_{j} * f \bigr)_{j} \bigr\Vert _{L^{p( \cdot)} ( \ell^{q( \cdot)} )}. $$
(ii) Let ${p},{q}\in\mathcal{P}_{0}^{\log} ( \mathbb{{R}}^{{n}} )$ and let ${s}\in{C}_{{\mathrm{loc}}}^{\log} ( \mathbb{{R}}^{{n}} )$.
  $$B_{p( \cdot),q( \cdot)}^{s( \cdot )}\bigl(\mathbb{R}^{n}\bigr): = \bigl\{ f \in\mathcal {{S}}'\bigl(\mathbb{R}^{n}\bigr): \Vert f \Vert _{B_{p( \cdot),q( \cdot )}^{s( \cdot)}}^{\varphi} < \infty \bigr\} , $$
  where
  $$\Vert f \Vert _{B_{p( \cdot),q( \cdot)}^{s( \cdot)}}^{\varphi}: = \bigl\Vert \bigl( 2^{ks( \cdot)}\varphi_{k} * f \bigr)_{k} \bigr\Vert _{\ell^{q( \cdot)} ( L^{p( \cdot)} )}. $$

The key tool will be the Peetre maximal operators, which were introduced by Peetre in [40]. Let *a* be a positive number and a system $( \Phi_{{k}} )_{{k}\in\mathbb{{N}}_{0}}$ in $\mathcal{S} ( \mathbb{{R}}^{{n}} )$. Then the Peetre maximal operators associated to $( \Phi_{{k}} )_{{k}\in \mathbb{{N}}_{0}}$ are defined by for each distribution ${f}\in \mathcal{S}' ( \mathbb{{R}}^{{n}} )$

$$\Phi_{k}^{ * a}f: = \sup_{y \in\mathbb{R}^{n}} \frac{ \vert ( \Phi_{k} * f ) ( y ) \vert }{1 + \vert 2^{k} ( y - x ) \vert ^{a}},\quad x \in\mathbb{R}^{n}\mbox{ and } k \in \mathbb{N}_{0} . $$

We start with two given functions $\phi_{0}, \phi_{1} \in\mathcal{S} ( \mathbb{{R}}^{{n}} )$. We define

$$\phi_{j} ( x ) = \phi_{1} \bigl( 2^{ - j + 1}x \bigr) \quad\mbox{for } x \in\mathbb{R}^{n}\mbox{ and } j \in \mathbb{N} . $$

Moreover, for each ${j}\in \mathbb{{N}}_{0}$, we denote $\Phi_{{j}} = \hat{\phi}_{{j}}$. We shall use the following result.

### Lemma 2.4

(Theorem 14 in [31])

*Let* ${p},{q}\in \mathcal{P}_{0}^{\log} ( \mathbb{{R}}^{{n}} )$ *with* ${p}^{+}, {q}^{+} < \infty$, *and* ${s} \in{C}_{{\mathrm{loc}}}^{\log} ( \mathbb{{R}}^{{n}} )$. *Let* ${R}\in\mathbb{{N}}_{0}$ *with* ${R} > {s}^{+}$ *and let* $\phi_{0}$, $\phi_{1}$ *belong to* $\mathcal{S} ( \mathbb{{R}}^{{n}} )$ *with*

$$D^{\beta} \phi_{1}(0) = 0\quad\textit{for } 0 \le \vert \beta \vert < R, $$

*and*

$$\begin{gathered} \bigl\vert \phi_{0}(x) \bigr\vert > 0\quad\textit{on } \bigl\{ x \in \mathbb{R}^{n}: \vert x \vert < \varepsilon \bigr\} , \\ \bigl\vert \phi_{1}(x) \bigr\vert > 0\quad\textit{on } \bigl\{ x \in \mathbb{R}^{n}:\varepsilon/ 2 < \vert x \vert < 2\varepsilon \bigr\} \end{gathered} $$

*for some* $\varepsilon>0$.

(i) *If* ${a} > \frac{{n}+ {C}_{\log} ( {1} / {{q}} )}{{p}^{-}} + {C}_{\log} ( {s} )$, *then*, *for all* ${f}\in\mathcal{S}' ( \mathbb{{R}}^{{n}} )$, *we have*
  1 $$ \Vert f \Vert _{B_{p( \cdot),q( \cdot)}^{s( \cdot)}} \approx \bigl\Vert \bigl\{ 2^{ks( \cdot)} ( \Phi_{k} * f ) \bigr\} _{k = 0}^{\infty} \bigr\Vert _{\ell^{q( \cdot)} ( L^{p( \cdot)} )} \approx \bigl\Vert \bigl\{ 2^{ks( \cdot)} \Phi_{k}^{ * a}f \bigr\} _{k = 0}^{\infty} \bigr\Vert _{\ell^{q( \cdot)} ( L^{p( \cdot)} )}. $$
(ii) *If* ${a} > \frac{{n}}{\min( {p}^{-}, {q}^{-} )} + {C}_{\log} ( {s} )$, *then*, *for all* ${f}\in \mathcal{S}' ( \mathbb{{R}}^{{n}} )$, *we have*
  2 $$ \Vert f \Vert _{F_{p( \cdot),q( \cdot)}^{s( \cdot )}} \approx \bigl\Vert \bigl\{ 2^{ks( \cdot)} ( \Phi_{k} * f ) \bigr\} _{k = 0}^{\infty} \bigr\Vert _{L^{p( \cdot)} ( \ell^{q( \cdot)} )} \approx \bigl\Vert \bigl\{ 2^{ks( \cdot)} \Phi_{k}^{ * a}f \bigr\} _{k = 0}^{\infty} \bigr\Vert _{L^{p( \cdot)} ( \ell^{q( \cdot)} )}. $$

Denote $\eta_{{v},{m}} := 2^{{nv}} (1+ 2^{{v}} | {x} | )^{-{m}}$, for ${v} \in\mathbb{{N}}_{0}$, ${m}\in\mathbb{{R}}$ and ${x}\in\mathbb{{R}}^{{n}}$.

### Lemma 2.5

(Lemma A.3 in [13])

*Let* ${v}_{0}, {v}_{1} \geq0$ *and* $m >n$. *Then*

$$\eta_{\nu_{0},m} * \eta_{\nu_{1},m} \approx\eta_{\min \{ \nu_{0},\nu_{1} \},m}. $$

Here the implicit constant depends only on *m* and *n*.

### Lemma 2.6

(Lemma A.6 in [13])

*Let* $r > 0$, ${v}\geq0$ *and* $m > n$. *Then there exists* $c > 0$, *which depends only on* *m*, *n* *and* *r*, *such that*, *for all* ${g}\in \mathcal{S}' ( \mathbb{{R}}^{{n}} )$ *with* $\operatorname{supp} \hat{ {g}} \subset \{ \xi\in \mathbb{{R}}^{{n}}: | \xi| \leq2^{{v}+1} \}$,

$$\bigl\vert g(x) \bigr\vert \le c \bigl( \eta_{\nu,m} * \vert g \vert ^{r}(x) \bigr)^{\frac{1}{r}},\quad x \in\mathbb{R}^{n}. $$

### Lemma 2.7

(Theorem 3.2 in [13])

*Let* ${p},{q}\in \mathcal{P}^{\log} ( \mathbb{{R}}^{{n}} )$ *with* $1 < {p}^{-} \leq{p}^{+} < \infty$ *and* $1 < {q}^{-} \leq{q}^{+} < \infty$. *Then the inequality*

$$\bigl\Vert ( \eta_{\nu,m} * f )_{\nu= 0}^{\infty} \bigr\Vert _{L^{p( \cdot)} ( \ell^{q( \cdot)} )} \le c \bigl\Vert ( f_{\nu} )_{\nu= 0}^{\infty} \bigr\Vert _{L^{p( \cdot)} ( \ell^{q( \cdot)} )} $$

*holds for every sequence* $( {f}_{{v}} )_{{v}\in\mathbb{{N}}_{0}} $ *of locally integrable functions and* $m > n$.

### Lemma 2.8

(Lemma 4.7 in [3])

*Let* ${p},{q} \in \mathcal{P}^{\log} ( \mathbb{{R}}^{{n}} )$ *with* $1 < {p}^{-} \leq{p}^{+} < \infty$ *and* $1 < {q}^{-} \leq{q}^{+} < \infty$. *For* $m > n$, *there exists* $c > 0$ *such that*

$$\bigl\Vert ( \eta_{\nu,2m} * f_{\nu} )_{\nu} \bigr\Vert _{\ell^{q( \cdot)} ( L^{p( \cdot)} )} \le c \bigl\Vert ( f_{\nu} )_{\nu} \bigr\Vert _{\ell^{q( \cdot)} ( L^{p( \cdot)} )}. $$

In Lemmas 2.7 and 2.8, we required that ${p}^{-}, {q}^{-} > 1$. This restriction can often be overcome by using Lemma 2.6 and the following identity:

$$\bigl\Vert ( f_{\nu} )_{\nu} \bigr\Vert _{\ell^{q( \cdot)} ( L^{p( \cdot)} )} = \bigl\Vert \bigl( \vert f_{\nu} \vert ^{r} \bigr)_{\nu} \bigr\Vert ^{\frac{1}{r}}_{\ell^{\frac{q( \cdot)}{r}} ( L^{\frac{p( \cdot)}{r}} )} $$

and

$$\bigl\Vert ( f_{\nu} )_{\nu} \bigr\Vert _{L^{p( \cdot)} ( \ell^{q( \cdot)} )} = \bigl\Vert \bigl( \vert f \vert _{\nu}^{r} \bigr)_{\nu} \bigr\Vert ^{\frac{1}{r}}_{L^{\frac{p( \cdot)}{r}} ( \ell^{\frac{q( \cdot)}{r}} )}. $$

### Lemma 2.9

(Lemma 6.1 in [13])

*If* ${s}({\cdot})\in{C}_{{\mathrm{loc}}}^{\log}$, *then there exists* ${t}\in ( {n},\infty )$ *such that if* $m>t$, *then*

$$2^{{vs} ( {\cdot} )} \eta_{{v},2{m}} ({x}-{y})\leq{c} 2^{{vs} ( {\cdot} )} \eta_{{v},{m}} ({x}-{y}) {,} $$

*with* $c>0$ *independent of* ${x}, {y}\in \mathbb{{R}}^{{n}}$ *and* ${v}\in \mathbb{{N}}_{{0}}$. *Therefore*,

$$2^{{vs} ( {\cdot} )} \eta_{{v},2{m}} *{f} ( {x} ) \leq {c} \eta_{{v},{m}} * \bigl( 2^{{vs} ( {\cdot} )} {f} \bigr) ({x}) {.} $$

### Lemma 2.10

(Lemma 9 in [31])

*Let* ${p},{q} \in\mathcal{P}_{0} ( \mathbb{{R}}^{{n}} )$ *and* $\delta>0$. *Let* $( {g}_{{k}} )_{{k}\in\mathbb{{Z}}} $ *be a sequence of non*-*negative measurable functions on* $\mathbb{{R}}^{{n}} $ *and define*

$$G_{\nu} ( x ): = \sum_{k \in\mathbb{Z}} 2^{ - |\nu- k|\delta} g_{k} ( x ),\quad x \in\mathbb{R}^{n},\nu\in \mathbb{R}^{n}. $$

*Then there exist constants* ${c}_{1}, {c}_{2} > 0$, *depending on* ${p} ( {\cdot} )$, ${q}({\cdot})$ *and* *δ*, *such that*

$$\begin{gathered} \Vert G_{\nu} \Vert _{\ell^{q( \cdot)} ( L^{p( \cdot)} )} \le c_{1} \Vert g_{k} \Vert _{\ell^{q( \cdot )} ( L^{p( \cdot)} )}, \\ \Vert G_{\nu} \Vert _{L^{p( \cdot)} ( \ell^{q( \cdot)} )} \le c_{2} \Vert g_{k} \Vert _{L^{p( \cdot)} ( \ell^{q( \cdot)} )}. \end{gathered} $$

### Lemma 2.11

(Theorem 3.6 in [3])

*Let* ${p},{q} \in\mathcal{P}$. *If either* $\frac{1}{ {p}} + \frac{1}{{q}} \leq1$ *pointwise*, *or* *q* *is a constant*, *then* $\Vert {\cdot} \Vert _{\ell^{{q}({\cdot})} ( {L}^{{p}({\cdot})} )}$ *is a norm*.

### Lemma 2.12

(Theorem 6.1 in [3])

*Let* ${s}, {s}_{0}, {s}_{1} \in{L}^{\infty}$ *and* ${p}, {q}_{0}, {q}_{1} \in\mathcal{P}_{0} $

(i) *If* ${q}_{0} \leq{q}_{1}$ *then* ${B}_{{p}({\cdot }), {q}_{0} ({\cdot})}^{{s}({\cdot})} \hookrightarrow {B}_{{p}({\cdot}), {q}_{1} ({\cdot})}^{{s}({\cdot})}$.
(ii) *If* $( {s}_{0} - {s}_{1} )^{-} > 0$, *then* ${B}_{{p}({\cdot}), {q}_{0} ({\cdot})}^{{s}_{0} ({\cdot})} \hookrightarrow {B}_{{p}({\cdot}), {q}_{1} ({\cdot})}^{{s}_{1} ({\cdot})}$.
(iii) *If* ${p}^{+}, {q}^{+} < \infty$, *then* ${B}_{{p}({\cdot}),\min \{ {p}({\cdot}),q({\cdot}) \}}^{{s}({\cdot})} \hookrightarrow {F}_{{p}({\cdot}),q({\cdot})}^{{s}( {\cdot})} \hookrightarrow{B}_{{p}({\cdot}),\max \{ {p}({\cdot}),q({\cdot}) \}}^{{s}({\cdot})}$.

### Remark 2.13

If ${p}\in\mathcal{P}^{\log} ( \mathbb{{R}}^{{n}} )$ with $1< p^{-}\le p^{+}<\infty$, then Theorem 12.5.7 in [12] says that ${F}_{{p} ( {\cdot} ),2}^{0} ( \mathbb{{R}}^{{n}} ) = {L}^{{p} ( {\cdot} )}$.

We shall use characterizations of ${B}_{{p} ( {\cdot} ),{q} ( {\cdot} )}^{{s} ( {\cdot} )} ( \mathbb{{R}}^{{n}} ) $ and ${F}_{{p} ( {\cdot} ),{q} ( {\cdot} )}^{{s} ( {\cdot} )} ( \mathbb{{R}}^{{n}} )$ by approximation, which are a generalization of the classical Besov and Triebel–Lizorkin spaces. For the latter, see [47].

Let

$$\Omega_{p( \cdot)} \bigl(\mathbb{R}^{n} \bigr) = \bigl\{ ( u_{\nu} )_{\nu} \subset\mathcal{{S}}' \bigl( \mathbb{R}^{n} \bigr) \cap L^{p( \cdot )} \bigl(\mathbb{R}^{n} \bigr):\operatorname{supp}\hat{u}_{\nu} \subset \bigl\{ \xi: \vert \xi \vert \le2^{\nu+ 1} \bigr\} ,\nu\in\mathbb{N}_{0} \bigr\} . $$

### Lemma 2.14

(Theorem 8.1 in [3])

*Let* ${p},{q} \in\mathcal{P}_{0}^{\log} ( \mathbb{{R}}^{{n}} )$ *and* ${s} \in {C}_{{\mathrm{loc}}}^{\log} \cap {L}^{\infty}$ *with* ${s}^{-} > 0$. *Let* ${f}\in\mathcal{S}' ( \mathbb{{R}}^{{n}} )$. *Then* *f* *is in* ${B}_{{p} ( {\cdot} ),{q} ( {\cdot} )}^{{s} ( {\cdot} )} $ *if and only if there exists* $\omega= ( \omega_{{j}} )_{{j}} \in \Omega_{{p}({\cdot})} ( \mathbb{{R}}^{{n}} )$ *such that* ${f}= \lim_{{k}\rightarrow\infty} \omega_{{k}}$ *in* $\mathcal{S}' ( \mathbb{{R}}^{{n}} )$ *and*

3 $$ \Vert f \Vert _{B_{p( \cdot),q( \cdot)}^{s( \cdot)}}^{\omega}: = \Vert \omega_{0} \Vert _{L^{p( \cdot)}} + \bigl\Vert \bigl( 2^{ks( \cdot )} ( f - \omega_{k} ) \bigr)_{k\in \mathbb{N}} \bigr\Vert _{\ell^{q( \cdot )} ( L^{p( \cdot)} )} < \infty. $$

*Furthermore*,

$$\Vert f \Vert _{B_{p( \cdot),q( \cdot)}^{s( \cdot)}}^{ *} = \inf \Vert f \Vert _{B_{p( \cdot),q( \cdot)}^{s( \cdot)}}^{\omega}, $$

*where the infimum is taken over all admissible systems* $\omega\in \Omega_{{p}({\cdot})} ( \mathbb{{R}}^{{n}} )$, *is an equivalent quasi*-*norm in* ${B}_{{p} ( {\cdot} ),{q} ( {\cdot} )}^{{s} ( {\cdot} )}$.

### Theorem 2.15

*Let* ${p},{q} \in \mathcal{P}_{0}^{\log} ( \mathbb{{R}}^{{n}} )$ *with* ${p}^{+} < \infty $ *and* ${s} \in {C}_{{\mathrm{loc}}}^{\log} \cap{L}^{\infty}$ *with* ${s}^{-} > 0$. *Then* ${f}\in \mathcal{S}' ( \mathbb{{R}}^{{n}} )$ *belongs to* ${F}_{{p} ( {\cdot} ),{q} ( {\cdot} )}^{{s} ( {\cdot} )} ( \mathbb{{R}}^{{n}} )$ *if and only if there exists* $\omega = ( \omega_{{j}} )_{{j}} \in \Omega_{{p}({\cdot})} ( \mathbb{{R}}^{{n}} )$ *such that* ${f}= \lim_{{j}\rightarrow\infty} \omega_{{j}}$ *in* $\mathcal{S}' ( \mathbb{{R}}^{{n}} )$ *and*

4 $$ \Vert f \Vert _{F_{p( \cdot),q( \cdot)}^{s( \cdot)}}^{\omega}: = \Vert \omega_{0} \Vert _{L^{p( \cdot)}} + \bigl\Vert \bigl( 2^{ks( \cdot )} ( f - \omega_{k} ) \bigr)_{k\in \mathbb{N}} \bigr\Vert _{L^{p( \cdot )}(\ell^{q( \cdot)})} < \infty. $$

*Furthermore*,

$$\Vert f \Vert _{F_{p( \cdot),q( \cdot)}^{s( \cdot)}}^{ *} = \inf \Vert f \Vert _{F_{p( \cdot),q( \cdot)}^{s( \cdot)}}^{\omega}, $$

*where the infimum is taken over all admissible systems* $\omega\in \Omega_{{p}({\cdot})} ( \mathbb{{R}}^{{n}} )$, *is an equivalent quasi*-*norm in* ${F}_{{p} ( {\cdot} ),{q} ( {\cdot} )}^{{s} ( {\cdot} )}$.

### Proof

First we show that there is a constant *C* independent of f such that

5 $$ \Vert f \Vert _{F_{p( \cdot),q( \cdot)}^{s( \cdot)}}^{ *} \le C \Vert f \Vert _{F_{p( \cdot),q( \cdot)}^{s( \cdot)}}. $$

Let $( \varphi_{{j}} )_{{j}}$ be functions in $\mathbb {{R}}^{{n}}$ as defined in Definition 2.2, then

$$\omega_{j}: = \sum_{k = 0}^{j} \varphi_{k} * f \to f\quad\mbox{in } \mathcal{{S}}' \bigl( \mathbb{R}^{n} \bigr) \mbox{ as } j \to\infty . $$

Thus $( \omega_{{j}} )_{{j}} \in \Omega_{{p}({\cdot})} ( \mathbb{{R}}^{{n}} )$ and

$$\bigl( 2^{js( \cdot)} ( f - \omega_{j} ) \bigr)_{j} = \sum_{k \ge0} 2^{ - ks( \cdot)} \bigl( 2^{(j + k)s( \cdot)} \varphi_{j + k} * f \bigr)_{j} $$

in $\mathcal{S}' ( \mathbb{{R}}^{{n}} )$. Notice that $2^{-{ks}({\cdot})} \leq2^{-{k} {s}^{-}}$ and that ${s}^{-} > 0$ by assumption. If ${q} ( {x} ) \in[1,\infty]$, by Minkowski’s inequality, we have

6 $$ \begin{aligned}[b] \Biggl( \sum_{j = 0}^{\infty} 2^{js(x)q(x)} \vert f - \omega_{j} \vert ^{q(x)} \Biggr)^{\frac{1}{q(x)}} &\le\sum_{k = 0}^{\infty} 2^{ - ks(x)} \Biggl( \sum_{j = 0}^{\infty} 2^{(j + k)s(x)q(x)} \bigl\vert ( \varphi_{j + k} * f ) (x) \bigr\vert ^{q(x)} \Biggr)^{\frac{1}{q(x)}} \\ &\le\sum_{k = 0}^{\infty} 2^{ - ks^{ -}} \Biggl( \sum_{j = 0}^{\infty} 2^{(j + k)s(x)q(x)} \bigl\vert ( \varphi_{j + k} * f ) (x) \bigr\vert ^{q(x)} \Biggr)^{\frac{1}{q(x)}} \\ &\le C \Biggl( \sum_{j = 0}^{\infty} 2^{js(x)q(x)} \bigl\vert ( \varphi _{j} * f ) (x) \bigr\vert ^{q(x)} \Biggr)^{\frac{1}{q(x)}} \end{aligned} $$

(modification if ${q} ( {x} ) =\infty$). If ${q} ( {x} ) \in(0,1)$, since ${q}^{-} > 0$, we have

7 $$ \begin{aligned}[b] \sum_{j = 0}^{\infty} 2^{js(x)q(x)} \vert f - \omega_{j} \vert ^{q(x)} &\le \sum_{k = 0}^{\infty} 2^{ - ks(x)q(x)} \sum _{j = 0}^{\infty} 2^{(j + k)s(x)q(x)} \bigl\vert ( \varphi_{j + k} * f ) (x) \bigr\vert ^{q(x)} \\ &\le\sum_{k = 0}^{\infty} 2^{ - ks^{ -} q(x)} \sum _{j = 0}^{\infty} 2^{(j + k)s(x)q(x)} \bigl\vert ( \varphi_{j + k} * f ) (x) \bigr\vert ^{q(x)} \\ &\le\sum_{k = 0}^{\infty} 2^{ - ks^{ -} q^{ -}} \sum _{j = 0}^{\infty} 2^{(j + k)s(x)q(x)} \bigl\vert ( \varphi_{j + k} * f ) (x) \bigr\vert ^{q(x)} \\ &\le C\sum_{j = 0}^{\infty} 2^{js(x)q(x)} \bigl\vert ( \varphi_{j} * f ) (x) \bigr\vert ^{q(x)}. \end{aligned} $$

Thus from (6) and (7), for ${q}\in \mathcal{P}_{0}$, we have

$$\Biggl( \sum_{j = 0}^{\infty} 2^{js ( x )q ( x )} \vert f - \omega_{j} \vert ^{q ( x )} \Biggr)^{\frac{1}{q ( x )}} \le C\sum_{j = 0}^{\infty} 2^{js ( x )q ( x )} \bigl\vert ( \varphi_{j} * f ) ( x ) \bigr\vert ^{q ( x )}. $$

Taking the ${L}^{{p}({\cdot})} ( \mathbb{{R}}^{{n}} )$-quasi-norm on the above inequality, we obtain (5) since

$$\Vert \omega_{0} \Vert _{L^{p( \cdot)}} = \Vert \varphi_{0} * f \Vert _{L^{p( \cdot)}} \le C \Vert f \Vert _{F_{p( \cdot),q( \cdot )}^{s( \cdot)}}. $$

Now we show the opposite inequality of (5). Let $( \omega_{{k}} )_{{k}} \in\Omega_{{p}({\cdot})} ( \mathbb{{R}}^{{n}} )$ such that ${f}= \lim_{{k}\rightarrow\infty} \omega_{{k}} $ and $\Vert {f} \Vert ^{\omega} < \infty$. Then $\varphi_{{j}} *{f}= \sum_{{k}=-1}^{\infty} \varphi_{{j}} * ( \omega_{{k}+{j}} - \omega_{{k}+{j}-1} )$, ${j}\in \mathbb{{N}}_{0}$ (with $\omega_{-1} =0$). Since

$$\vert \varphi_{j} * f \vert = \Biggl\vert \sum _{k = - 1}^{\infty} \varphi_{j} * ( \omega_{k + j} - \omega_{k + j - 1} ) \Biggr\vert \le\sum _{k = - 1}^{\infty} \bigl\vert \varphi_{j} * ( \omega_{k + j} - \omega_{k + j - 1} ) \bigr\vert . $$

Let ${r}\in ( 0,\min \{ {p}^{-}, {q}^{-},1 \} )$. By the definition of $\varphi_{{j}}$, there exists a constant C > 0 such that $| \varphi_{{j}} | \leq C \eta_{{j}, \frac{2{m}}{{r}}}$, and using Lemma 2.6, then we conclude that

$$\bigl\vert \varphi_{j} * ( \omega_{k + j} - \omega_{k + j - 1} ) \bigr\vert \le C\eta_{j,\frac{2m}{r}} * \vert \omega_{k + j} - \omega_{k + j - 1} \vert \le C\eta_{j,\frac{2m}{r}} * \bigl( \eta_{k + j,2m} * \vert \omega_{k + j} - \omega_{k + j - 1} \vert ^{r} \bigr)^{\frac{1}{r}}. $$

By Minkowski’s integral inequality (with exponent $\frac{1}{{r}} > 1$) and Lemma 2.5 we obtain

$$\bigl\vert \varphi_{j} * ( \omega_{k + j} - \omega_{k + j - 1} ) \bigr\vert ^{r} \le C \bigl[ \eta_{j,\frac{2m}{r}} * \eta_{k + j,2m}^{\frac{1}{r}} \bigr]^{r} * \vert \omega_{k + j} - \omega_{k + j - 1} \vert ^{r} \le C \eta_{j,2m} * \vert \omega_{k + j} - \omega_{k + j - 1} \vert ^{r}. $$

Hence, by Lemma 2.9,

$$\begin{aligned} \bigl\vert 2^{js( \cdot)}\varphi_{j} * f ( x ) \bigr\vert & \le C\sum_{k = - 1}^{\infty} \bigl(\eta_{j,m} * 2^{js( \cdot)r} \vert \omega_{k + j} - \omega_{k + j - 1} \vert ^{r} \bigr)^{\frac{1}{r}} ( x ) \\ &\le C\sum_{k = - 1}^{\infty} 2^{ - ks^{ -}} \bigl( \eta_{j,m} * 2^{(j + k)s( \cdot)r} \vert \omega_{k + j} - \omega_{k + j - 1} \vert ^{r} \bigr)^{\frac{1}{r}} ( x ), \end{aligned} $$

Since $2^{{js}({\cdot})} \leq 2^{({j}+{k}){s}({\cdot})} 2^{-{k} {s}^{-}}$. So we have

$$\begin{aligned} \bigl\Vert \bigl( 2^{js( \cdot)} \varphi_{j} * f \bigr)_{j} \bigr\Vert _{L^{p( \cdot)}(\ell^{q( \cdot)})} &= \bigl\Vert \bigl( \bigl\vert 2^{js( \cdot)}\varphi_{j} * f \bigr\vert ^{r} \bigr)_{j} \bigr\Vert ^{\frac{1}{r}}_{L^{\frac{p( \cdot)}{r}}(\ell^{\frac{q( \cdot )}{r}})} \\ &\le C \Biggl\Vert \Biggl( \sum_{k = - 1}^{\infty} 2^{ - krs^{ -}} \bigl( \eta_{j,m} * 2^{(j + k)s( \cdot)r} \vert \omega_{k + j} - \omega_{k + j - 1} \vert ^{r} \bigr) \Biggr)_{j} \Biggr\Vert ^{\frac{1}{r}}_{L^{\frac{p( \cdot)}{r}}(\ell^{\frac{q( \cdot)}{r}})} \\ &\le C\sum_{k = - 1}^{\infty} 2^{ - ks^{ -}} \bigl\Vert \bigl( \eta_{j,m} * 2^{(j + k)s( \cdot)r} \vert \omega_{k + j} - \omega_{k + j - 1} \vert ^{r} \bigr)_{j} \bigr\Vert ^{\frac{1}{r}}_{L^{\frac{p( \cdot )}{r}}(\ell^{\frac{q( \cdot)}{r}})} \\ &\le C\sum_{k = - 1}^{\infty} 2^{ - ks^{ -}} \bigl\Vert \bigl( 2^{(j + k)s( \cdot)} ( \omega_{k + j} - \omega_{k + j - 1} ) \bigr)_{j} \bigr\Vert _{L^{p( \cdot)}(\ell^{q( \cdot)})}, \end{aligned} $$

where the last inequality is due to Lemma 2.7. Now using $\vert \omega_{{k}+{j}} - \omega_{{k}+{j}-1} \vert \leq \vert {f}- \omega_{{k}+{j}} \vert + \vert {f}- \omega_{{k}+{j}-1} \vert $, we find that

$$\begin{aligned} \bigl\Vert \bigl( 2^{js( \cdot)} \varphi_{j} * f \bigr)_{j} \bigr\Vert _{L^{p( \cdot)}(\ell^{q( \cdot)})} &\le C\sum_{k \ge- 1} 2^{ - ks^{ -}} \bigl\Vert \bigl( 2^{(j + k)s( \cdot)} ( f - \omega_{k + j} ) \bigr)_{j} \bigr\Vert _{L^{p( \cdot)}(\ell^{q( \cdot)})} \\ &\le C \bigl\Vert \bigl( 2^{(j + k)s( \cdot)} ( f - \omega_{k + j} ) \bigr)_{j} \bigr\Vert _{L^{p( \cdot)}(\ell^{q( \cdot)})}. \end{aligned} $$

Since the sequence space is invariant with respect to shifts, we arrive at that the left-hand side can be estimated by a constant times $\Vert {f} \Vert ^{\omega}$. Taking the infimum over *ω*, we conclude that $\Vert {f} \Vert _{ {F}_{{p} ( {\cdot} ),{q} ( {\cdot} )}^{{s} ( {\cdot} )}} \leq C \Vert {f} \Vert ^{*}$. □

The following generalized Hölder inequality will often be used in the sequel. It is Theorem 2.3 in [25].

### Lemma 2.16

*Let* ${p}, {p}_{1}, {p}_{2} \in \mathcal{P}_{0} ( \mathbb{{R}}^{{n}} )$ *with* ${p}_{1}^{+}, {p}_{2}^{+} < \infty$ *such that* $\frac{1}{{p}({x})} = \frac{1}{ {p}_{1({x})}} + \frac{1}{{p}_{2({x})}}$. *Then there exists a constant* ${C}_{{p}, {p}_{1}}$ *independent of the functions f and g such that*

$$\Vert fg \Vert _{L^{p ( \cdot )}} \le C_{p,p_{1}} \Vert f \Vert _{L^{p_{1} ( \cdot )}} \Vert g \Vert _{L^{p_{2} ( \cdot )}} $$

*holds for every* ${f}\in{L}^{{p}_{1} ({\cdot})}$ *and* ${g}\in{L}^{{p}_{2} ({\cdot})}$.

### Lemma 2.17

*Let* ${p}, {p}_{1}, {p}_{2} \in \mathcal{P}_{0} ( \mathbb{{R}}^{{n}} )$ *with* ${p}_{1}^{+}, {p}_{2}^{+} < \infty$ *such that* $\frac{1}{ {p}({x})} = \frac{1}{{p}_{1({x})}} + \frac{1}{ {p}_{2({x})}}$. *Then there is a constant* $C> 0$ *such that*, *for each* $\{ {f}_{{k}} \}_{{k}=0}^{\infty} \in \ell^{{q}({\cdot})} ( {L}^{{p}_{1} ({\cdot})} )$, ${h} \in {L}^{{p}_{2} ({\cdot})} $,

$$\bigl\Vert \{ f_{k}h \}_{k = 0}^{\infty} \bigr\Vert _{\ell^{q( \cdot )}(L^{p( \cdot)})} \le C \bigl\Vert \{ f_{k} \}_{k = 0}^{\infty} \bigr\Vert _{\ell^{q( \cdot)}(L^{p_{1}( \cdot)})} \Vert h \Vert _{L^{p_{2}( \cdot)}}. $$

### Proof

By a scaling argument, it suffices to consider the case $\Vert \{ {f}_{{k}} \}_{{k}=0}^{\infty} \Vert _{\ell^{{q}({\cdot})} ( {L}^{{p}_{1} ({\cdot})} )} =1$, $\Vert {h} \Vert _{{L}^{{p}_{2} ({\cdot})}} =1$. Let *C* is the constant in Lemma 2.16 for exponents $\frac{{p} ( {\cdot} )}{{q} ( {\cdot} )}$, $\frac{{p}_{1} ( {\cdot} )}{{q} ( {\cdot} )}$, $\frac{{p}_{2} ( {\cdot} )}{{q} ( {\cdot} )}$. So by Lemma 2.1.14 in [12], we have $\sum_{{k}=0}^{\infty} \Vert \vert {f}_{{k}} \vert ^{{q}({\cdot})} \Vert _{\frac{{p}_{1} ({\cdot})}{{q}({\cdot})}} =1$ and $\Vert \vert {h} \vert ^{{q}({\cdot})} \Vert _{\frac{{p}_{2} ({\cdot})}{{q}({\cdot})}} =1$. Using the generalized Hölder inequality (Lemma 2.16), then we have

$$\begin{aligned} \rho_{\ell^{q( \cdot)}(L^{p( \cdot)})} \biggl( \frac{1}{C} \{ f_{k}h \}_{k = 0}^{\infty} \biggr) &= \sum _{k = 0}^{\infty} \frac{1}{C}\big\| | f_{k}h |^{q( \cdot)} \big\| _{\frac{p( \cdot)}{q( \cdot)}} \\ &\le\sum_{k = 0}^{\infty} \big\| | f_{k} |^{q( \cdot)} \big\| _{\frac{p_{1}( \cdot)}{q( \cdot)}}\big\| | h |^{q( \cdot)} \big\| _{\frac{p_{2}( \cdot)}{q( \cdot)}} \\ &\le1. \end{aligned} $$

Thus $\Vert \{ {f}_{{k}} {h} \}_{{k}=0}^{\infty} \Vert _{\ell^{{q}({\cdot})} ( {L}^{{p}({\cdot})} )} \leq{C}$. □

## Main results

### Theorem 3.1

*Let* ${p}, {p}_{1}, {p}_{2}, q \in \mathcal{P}_{0}^{\log} ( \mathbb{{R}}^{{n}} )$ *such that* $1< p^{-},p_{1}^{-},p_{2}^{-}$, *and* ${p}_{1}^{+}, {p}_{2}^{+}, {p}^{+} ,q^{+}< \infty$ *and* $\frac{1}{{p}({x})} = \frac{1}{ {p}_{1({x})}} + \frac{1}{{p}_{2({x})}}$. *Let* ${s} \in {C}_{{\mathrm{loc}}}^{\log} \cap {L}^{\infty}$ *with* ${s}^{-} > 0$, *and* ${N},{M}\in\mathbb {{N}}_{0}$ *be even numbers with* ${N} > {s}^{+}$ *and* ${M} > \frac{{n}}{\min( {p}_{1}^{-}, {p}_{2}^{-}, {q}^{-} )} + {C}_{\log} ( {s} ) +{n}$. *Then there exists a positive constant* *C* *depending on* *N*, *M*, *n*, *p*, *q*, *s* *such that*

8 $$ \bigl\Vert T_{\sigma} ( f,g ) \bigr\Vert _{F_{p( \cdot),q( \cdot )}^{s( \cdot)}} \le C \Vert \sigma \Vert _{N,M} \bigl( \Vert f \Vert _{F_{p_{1}( \cdot),q( \cdot)}^{s( \cdot)}} \Vert g \Vert _{F_{p_{2}( \cdot),1}^{0}} + \Vert f \Vert _{F_{p_{1}( \cdot ),1}^{0}} \Vert g \Vert _{F_{p_{2}( \cdot),q( \cdot)}^{s( \cdot)}} \bigr) $$

*for every* ${f},{g} \in\mathcal{S} ( \mathbb{{R}}^{{n}} )$ *and* $\sigma\in {B} \mathcal{S}_{1,1}^{0}$. *Moreover*,

9 $$ \bigl\Vert T_{\sigma} ( f,g ) \bigr\Vert _{F_{p( \cdot),q( \cdot )}^{s( \cdot)}} \le C \Vert \sigma \Vert _{N,M} \Vert f \Vert _{F_{p_{1}( \cdot),q( \cdot)}^{s( \cdot)}} \Vert g \Vert _{F_{p_{2}( \cdot),q( \cdot)}^{s( \cdot)}} $$

*for every* ${f},{g} \in\mathcal{S} ( \mathbb{{R}}^{{n}} )$ *and* $\sigma\in {B} \mathcal{S}_{1,1}^{0}$.

### Theorem 3.2

*Let* ${p}, {p}_{1}, {p}_{2}, q \in \mathcal{P}_{0}^{\log} ( \mathbb{{R}}^{{n}} )$ *such that* $1< p^{-},p_{1}^{-},p_{2}^{-}$, *and* ${p}_{1}^{+}, {p}_{2}^{+}, {p}^{+} ,q^{+}< \infty$ *and* $\frac{1}{{p}({x})} = \frac{1}{ {p}_{1({x})}} + \frac{1}{{p}_{2({x})}}$. *Let* ${s} \in {C}_{{\mathrm{loc}}}^{\log} \cap{L}^{\infty} $ *with* ${s}^{-} > 0$, *and* ${N}, {M}\in \mathbb{{N}}_{0}$ *be even numbers with* ${N} > {s}^{+}$ *and* ${M} > \frac{{n}+ {C}_{\log} ( {1} / {{q}} )}{ \min( {p}_{1}^{-}, {p}_{2}^{-} )} + {C}_{\log} ( {s} ) +{n}$. *Then there exists a positive constant* *C* *depending on* *N*, *M*, *n*, *p*, *q*, *s* *such that*

10 $$ \bigl\Vert T_{\sigma} ( f,g ) \bigr\Vert _{B_{p( \cdot),q( \cdot )}^{s( \cdot)}} \le C \Vert \sigma \Vert _{N,M} \bigl( \Vert f \Vert _{B_{p_{1}( \cdot),q( \cdot)}^{s( \cdot)}} \Vert g \Vert _{F_{p_{2}( \cdot),1}^{0}} + \Vert f \Vert _{F_{p_{1}( \cdot ),1}^{0}} \Vert g \Vert _{B_{p_{2}( \cdot),q( \cdot)}^{s( \cdot)}} \bigr) $$

*for every* ${f},{g} \in\mathcal{S} ( \mathbb{{R}}^{{n}} )$ *and* $\sigma\in {B} \mathcal{S}_{1,1}^{0}$. *Moreover*,

11 $$ \bigl\Vert T_{\sigma} ( f,g ) \bigr\Vert _{B_{p( \cdot),q( \cdot )}^{s( \cdot)}} \le C \Vert \sigma \Vert _{N,M} \Vert f \Vert _{B_{p_{1}( \cdot),q( \cdot)}^{s( \cdot)}} \Vert g \Vert _{B_{p_{2}( \cdot),q( \cdot)}^{s( \cdot)}} $$

*for every* ${f},{g} \in\mathcal{S} ( \mathbb{{R}}^{{n}} )$ *and* $\sigma\in {B} \mathcal{S}_{1,1}^{0}$.

To prove Theorems 3.1 and 3.2, we shall decompose the symbol function *σ* as usual, indeed, we shall follow the method in [37].

Let ${f},{g} \in\mathcal{S} ( \mathbb{{R}}^{{n}} )$ and $\{ \hat{\varphi}_{{k}} \}_{{k}\in \mathbb{{N}}_{0}}$ be functions in $\mathbb{{R}}^{{n}}$ as Definition 2.2. We write

$$\begin{aligned} T_{\sigma} (f,g) ( x )& = \int_{\mathbb{R}^{2n}} \sigma ( x,\xi,\eta ) \widehat{f} ( \xi )\widehat{g} ( \eta )e^{2\pi ix ( \xi+ \eta )}\,{d}\xi\,{d}\eta \\ &= \sum_{j,k \in\mathbb{N}_{0}} \int_{\mathbb{R}^{2n}} \sigma ( x,\xi,\eta ) \widehat{\varphi}_{k} ( \xi )\widehat{\varphi}_{j} ( \eta )\widehat{f} ( \xi )\widehat{g} ( \eta )e^{2\pi ix ( \xi+ \eta )}\,{d}\xi\,{d}\eta \\ &= \sum_{j,k \in\mathbb{N}_{0}} \int_{\mathbb{R}^{2n}} \int_{\mathbb{R}^{n}} \widehat{\sigma}^{1} ( \zeta,\xi,\eta ) e^{2\pi ix\zeta} \,{d}\zeta\widehat{\varphi}_{k} ( \xi )\widehat{ \varphi}_{j} ( \eta )\widehat{f} ( \xi )\widehat{g} ( \eta )e^{2\pi ix ( \xi+ \eta )}\,{d}\xi\,{d}\eta \\ &=:I_{1} ( x ) + I_{2} ( x ), \end{aligned} $$

where $\hat{\sigma}^{1} ( {\cdot},{\cdot},{\cdot} )$ stands for the Fourier transform of $\sigma ( {\cdot},{\cdot},{\cdot} )$ with respect to the first variable and

$$\begin{gathered} I_{1} ( x ): = \sum _{j,k \in\mathbb{N}_{0},j \le k} \int_{\mathbb{R}^{2n}} \int_{\mathbb{R}^{n}} \widehat{\sigma}^{1} ( \zeta,\xi,\eta ) e^{2\pi ix\zeta}\, {d}\zeta\widehat{\varphi}_{k} ( \xi )\widehat{ \varphi}_{j} ( \eta )\widehat{f} ( \xi )\widehat{g} ( \eta )e^{2\pi ix ( \xi+ \eta )}\,{d}\xi\,{d}\eta, \\ I_{2} ( x ): = \sum_{j,k \in\mathbb{N}_{0},k < j} \int_{\mathbb{R}^{2n}} \int_{\mathbb{R}^{n}} \widehat{\sigma}^{1} ( \zeta,\xi,\eta ) e^{2\pi ix\zeta}\, {d}\zeta \widehat{\varphi}_{k} ( \xi )\widehat{ \varphi}_{j} ( \eta )\widehat{f} ( \xi )\widehat{g} ( \eta )e^{2\pi ix ( \xi+ \eta )}\,{d}\xi\,{d}\eta. \end{gathered} $$

Now we need only to estimate ${I}_{1}$, since the estimate for ${I}_{2}$ will follow from the one for ${I}_{1}$ by interchanging the roles of *k* and *j*, *f* and *g*, and *ξ* and *η*. Using a partition of unity with respect to the variable *ζ* we write

12 $$ I_{1} ( x ) = \sum_{\ell\in\mathbb {N}_{0}}^{\infty} \sum_{j,k \in\mathbb{N}_{0},j \le k} T_{\sigma_{j,k,\ell}} (f,g) ( x ), $$

where the symbols are given by

$$\begin{gathered} \sigma_{j,k,0} ( x,\xi,\eta ): = \widehat{ \varphi}_{k} ( \xi )\widehat{\varphi}_{j} ( \eta ) \int_{\mathbb{R}^{n}} \Biggl( \sum_{\nu= 0}^{k} \widehat{\varphi}_{\nu} ( \zeta ) \Biggr)\widehat{\sigma}^{1} ( \zeta,\xi,\eta ) e^{2\pi ix\zeta} \,{d}\zeta, \\ \sigma_{j,k,\ell} ( x,\xi,\eta ): = \widehat{\varphi}_{k} ( \xi )\widehat{\varphi}_{j} ( \eta ) \int_{\mathbb{R}^{n}} \widehat{\varphi}_{k + \ell} ( \zeta )\widehat{ \sigma}^{1} ( \zeta,\xi,\eta ) e^{2\pi ix\zeta} \,{d}\zeta, \quad\ell \ge1 \end{gathered} $$

for $j\leq k$. For $\sigma_{{j},{k}, \ell}$ we use the following estimates.

### Lemma 3.3

(Lemma 3.1 in [37])

*If* ${N}, {M}\in \mathbb{{N}}_{0}$, *with N even*, *and multi*-*indices* $\alpha,\beta\in\mathbb{{N}}_{0}^{{n}}$ *satisfy* $\vert \alpha \vert + \vert \beta \vert \leq{M} $, *then there is a constant* *C* *depending only on* *M*, *N* *and* *n* *such that*

$$\bigl\vert \partial_{\xi}^{\alpha} \partial_{\eta}^{\beta} \sigma_{j,k,\ell} (x,\xi,\eta) \bigr\vert \le C \Vert \sigma \Vert _{N,M}2^{ - \ell n - k \vert \alpha \vert - j \vert \beta \vert }. $$

*for all* ${x}, \xi, \eta\in\mathbb{{R}}^{{n}}$, ${j}, {k}, \ell\in\mathbb{{N}}_{0}$, ${j}\leq{k}$, $\sigma\in{B} \mathcal{S}_{1,1}^{0}$.

### Lemma 3.4

(Lemma 3.2 in [37])

*Let*

13 $$ Q_{j,k,\ell} ( x,y,z ): = \bigl( \mathcal {{F}}_{2n} \sigma_{j,k,\ell} ( x, \cdot, \cdot ) \bigr) ( y,z ), \quad x,y,z \in \mathbb{R}^{2n}, $$

*where* $\mathcal{F}_{2{n}} \sigma_{{j},{k}, \ell} ({x},{\cdot},{\cdot})$ *denotes the Fourier transform in* $\mathbb{{R}}^{2{n}}$ *of* $\sigma_{{j},{k}, \ell} ({x},{\cdot},{\cdot})$ *with respect to the last two variables*. *If* $a > 0$ *and* ${N}, {M}\in\mathbb{{N}}_{0}$ *are even with* $M > a + n$, *then there is a constant* *C* *depending only on* *M*, *N*, *a* *and* *n* *such that*

$$\int_{\mathbb{R}^{2n}} \bigl\vert Q_{j,k,\ell} ( x,y,z ) \bigr\vert \bigl( 1 + 2^{k} \vert y \vert + 2^{j} \vert z \vert \bigr)^{a}\,dy\,dz \le C \Vert \sigma \Vert _{N,M}2^{ - \ell N}, $$

*for all* ${x} \in\mathbb{{R}}^{{n}}$, ${j}, {k}, \ell\in\mathbb{{N}}_{0}$ *with* ${j}\leq{k}$, $\sigma\in{B} \mathcal{S}_{1,1}^{0}$.

### Lemma 3.5

*Let* ${s} \in {C}_{{\mathrm{loc}}}^{\log} \cap{L}^{\infty}$. *Let* ${p}, {p}_{1}, {p}_{2}, q \in \mathcal{P}_{0}^{\log} ( \mathbb{{R}}^{{n}} )$ *such that* $1< p^{-},p_{1}^{-},p_{2}^{-}$, *and* ${p}_{1}^{+}, {p}_{2}^{+}, {p}^{+} ,q^{+}< \infty$ *and* $\frac{1}{{p}({x})} = \frac{1}{ {p}_{1({x})}} + \frac{1}{{p}_{2({x})}}$. *Let* ${N}, {M}\in \mathbb{{N}}_{0}$ *be even numbers*.

- *If* ${M} > \frac{{n}}{\min ( {p}_{1}^{-}, {p}_{2}^{-}, {q}^{-} )} + {C}_{\log} ( {s} ) +{n}$, *then there exists a positive constant* *C* *depending on* *N*, *M*, *n*, *p*, *q*, *s* *such that*
  14 $$ \Biggl\Vert \Biggl\{ 2^{ks ( \cdot )}\sum_{j = 0}^{k} \bigl\vert {T}_{\sigma_{j,k,\ell}} ({f},{g}) \bigr\vert \Biggr\} _{k \in\mathbb{N}_{0}} \Biggr\Vert _{L^{p( \cdot)}(\ell^{q( \cdot)})} \le C \Vert \sigma \Vert _{N,M}2^{ - \ell N} \Vert f \Vert _{F_{p_{1}( \cdot),q( \cdot)}^{s( \cdot)}} \Vert g \Vert _{F_{p_{2}( \cdot ),1}^{0}} $$
  *for all* $\ell\in\mathbb{{N}}_{0}$, ${f}, {g} \in\mathcal{S} ( \mathbb{{R}}^{{n}} )$ *and* $\sigma\in{B} \mathcal{S}_{1,1}^{0}$.
- *If* ${M} > \frac{{n}+ {C}_{\log} ( {1} / {{q}} )}{\min ( {p}_{1}^{-}, {p}_{2}^{-} )} + {C}_{\log} ( {s} ) +{n}$, *then there exists a constant* *C* *depending only on* *N*, *M*, *n* *and* *p*, *q*, *s* *such that*
  15 $$ \Biggl\Vert \Biggl\{ 2^{ks ( \cdot )}\sum_{j = 0}^{k} \bigl\vert {T}_{\sigma_{j,k,\ell}} ({f},{g}) \bigr\vert \Biggr\} _{k \in\mathbb{N}_{0}} \Biggr\Vert _{\ell^{q( \cdot)}(L^{p( \cdot)})} \le C \Vert \sigma \Vert _{N,M}2^{ - \ell N} \Vert f \Vert _{B_{p_{1}( \cdot),q( \cdot)}^{s( \cdot)}} \Vert g \Vert _{F_{p_{2}( \cdot ),1}^{0}} $$
  *for all* $\ell\in\mathbb{{N}}_{0}$, ${f}, {g} \in\mathcal{S} ( \mathbb{{R}}^{{n}} )$ *and* $\sigma\in{B} \mathcal{S}_{1,1}^{0}$.

### Proof

Let $\{ \hat{\varphi}_{{k}} \}_{{k}\in\mathbb{{N}}_{0}}$ and $\{ \hat{\Psi}_{{k}} \}_{{k}\in\mathbb{{N}}_{0}}$ be functions in $\mathbb{{R}}^{{n}}$ as Definition 2.2 and put ${f}_{{k}} := \hat {\Psi}_{{k}} ({D}){f}$ and ${g}_{{j}} := \hat{\Psi}_{{j}} ({D}){g}$ for ${j}, {k} \in\mathbb{{N}}_{0}$, ${j}\leq {k}$. Then

$$\sigma_{j,k,\ell} ( x,\xi,\eta )\widehat{f} ( \xi )\widehat{g} ( \eta ) = \sigma_{j,k,\ell} ( x,\xi ,\eta )\widehat{f} ( \xi )\widehat{g} ( \eta )\hat{ \Psi}_{k} ( \xi )\hat{\Psi}_{j} ( \eta ) = \sigma_{j,k,\ell} ( x,\xi,\eta )\widehat{f_{k}} ( \xi ) \widehat{g_{j}} ( \eta ), $$

and we write

$${T}_{\sigma_{j,k,\ell}} ({f},{g}) ( x ) = \int_{\mathbb{R}^{2n}} \sigma_{j,k,\ell} ( x,\xi,\eta ) \widehat{f}_{k} ( \xi )\widehat{g}_{j} ( \eta )e^{2\pi ix ( \xi+ \eta )}\,{d}\xi\,{d}\eta. $$

From (13), for $a > 0$ with $M > a + n$, we have

$$\begin{gathered} \biggl\vert \int_{\mathbb{R}^{2n}} \sigma_{j,k,\ell} ( x,\xi,\eta ) \widehat{f_{k}} ( \xi )\widehat{g_{j}} ( \eta )e^{2\pi ix ( \xi+ \eta )}\,{d}\xi \,{d}\eta \biggr\vert \\ \quad= \biggl\vert \int_{\mathbb{R}^{2n}} Q_{j,k,\ell} ( x, - y, - z ) f_{k} ( x - y )g_{j} ( x - z )\,{d}y\,{d}z \biggr\vert \\ \quad= \biggl\vert \int_{\mathbb{R}^{2n}} Q_{j,k,\ell} ( x, - y, - z ) \bigl( 1 + 2^{k} \vert y \vert + 2^{j} \vert z \vert \bigr)^{a}\frac{f_{k} ( x - y )g_{j} ( x - z )}{ ( 1 + 2^{k} \vert y \vert + 2^{j} \vert z \vert )^{a}} \,{d}y\,{d}z \biggr\vert \\ \quad\le \int_{\mathbb{R}^{2n}} \bigl\vert Q_{j,k,\ell} ( x, - y, - z ) \bigr\vert \bigl( 1 + 2^{k} \vert y \vert + 2^{j} \vert z \vert \bigr)^{a}\frac{ \vert f_{k} ( x - y ) \vert \vert g_{j} ( x - z ) \vert }{ ( 1 + 2^{k} \vert y \vert )^{\frac{a}{2}} ( 1 + 2^{j} \vert z \vert )^{\frac{a}{2}}} \\ \quad\le C \Vert \sigma \Vert _{N,M}2^{ - \ell N} \Psi_{k}^{ * \frac{a}{2}}f ( x )\Psi_{j}^{ * \frac{a}{2}}g ( x ) \end{gathered} $$

for all ${x} \in\mathbb{{R}}^{{n}}$. In the last inequality we used Lemma 3.4 and the definition of the Peetre maximal operator. Thus, we obtain, for all ${x} \in\mathbb{{R}}^{{n}}$,

$$\big| {T}_{\sigma_{j,k,\ell}} ({f},{g}) ( x ) \big| \le C \Vert \sigma \Vert _{N,M}2^{ - \ell N}\Psi _{k}^{ * \frac{a}{2}}f ( x ) \Psi_{j}^{ * \frac{a}{2}}g ( x ). $$

To prove (14), after adding in $j \leq k$, multiplying by $2^{{ks}({\cdot})}$, we obtain

16 $$ 2^{ks ( x )}\sum_{j = 0}^{k} \big| {T}_{\sigma_{j,k,\ell}} ({f},{g}) ( x ) \big| \le C \Vert \sigma \Vert _{N,M}2^{ - \ell N}2^{ks ( x )}\Psi_{k}^{ * \frac{a}{2}}f ( x )\sum_{j = 0}^{k} \Psi_{j}^{ * \frac{a}{2}}g ( x ). $$

Then taking the $\ell_{{q}({\cdot})}$-norm in *k* we obtain

$$\begin{gathered} \Biggl( \sum_{k \in\mathbb{N}_{0}} \Biggl( 2^{ks ( x )}\sum _{j = 0}^{k} \bigl\vert {T}_{\sigma_{j,k,\ell}} ({f},{g}) ( x ) \bigr\vert \Biggr)^{q ( x )} \Biggr)^{\frac{1}{q ( x )}} \\\quad\le C \Vert \sigma \Vert _{N,M}2^{ - \ell N} \biggl( \sum _{k \in \mathbb{N}_{0}} \bigl( 2^{ks ( \cdot )}\Psi_{k}^{ * \frac{a}{2}}f ( x ) \bigr)^{q ( \cdot )} \biggr)^{\frac{1}{q ( \cdot )}}\sum _{j \in\mathbb{N}_{0}} \Psi_{j}^{ * \frac{a}{2}}g ( x ).\end{gathered} $$

Since ${M}-{n} > \frac{{n}}{\min ( {p}_{1}^{-}, {p}_{2}^{-}, {q}^{-} )} + {C}_{\log} ( {s} )$ by the assumption in item (a), we choose ${a} > \frac{{n}}{\min ( {p}_{1}^{-}, {p}_{2}^{-}, {q}^{-} )} + {C}_{\log} ( {s} )$ such that $M - n > a$. Using the generalized Hölder inequality and Lemma 2.4, we have

$$\begin{gathered} \Biggl\Vert \Biggl\{ 2^{ks ( \cdot )}\sum _{j = 0}^{k} \bigl\vert T_{\sigma_{j,k,\ell}} (f,g) \bigr\vert \Biggr\} _{k \in\mathbb{N}_{0}} \Biggr\Vert _{L^{p( \cdot)}(\ell^{q( \cdot)})} \\\quad\le C \Vert \sigma \Vert _{N,M}2^{ - \ell N} \biggl\Vert \biggl( \sum _{k \in\mathbb{N}_{0}} \bigl( 2^{ks ( \cdot )}\Psi_{k}^{ * \frac{a}{2}}f \bigr)^{q ( \cdot )} \biggr)^{\frac{1}{q ( \cdot )}}\sum_{j \in\mathbb{N}_{0}} \Psi_{j}^{ * \frac{a}{2}}g \biggr\Vert _{L^{p ( \cdot )}} \\ \quad\le C \Vert \sigma \Vert _{N,M}2^{ - \ell N} \biggl\Vert \biggl( \sum_{k \in \mathbb{N}_{0}} \bigl( 2^{ks ( \cdot )} \Psi_{k}^{ * \frac{a}{2}}f \bigr)^{q ( \cdot )} \biggr)^{\frac{1}{q ( \cdot )}} \biggr\Vert _{L^{p_{1} ( \cdot )}} \biggl\Vert \sum_{j \in\mathbb{N}_{0}} \Psi_{j}^{ * \frac{a}{2}}g \biggr\Vert _{L^{p_{2} ( \cdot )}} \\ \quad\le C \Vert \sigma \Vert _{N,M}2^{ - \ell N} \Vert f \Vert _{F_{p_{1}( \cdot),q( \cdot)}^{s( \cdot)}} \Vert g \Vert _{F_{p_{2}( \cdot),1}^{0}}. \end{gathered} $$

This is the inequality (14).

To prove (b), from above inequality (16), taking $\ell^{{q}({\cdot})} ( {L}^{{p}({\cdot})} )$-norm in *k*, we have

$$\begin{gathered} \Biggl\Vert \Biggl\{ 2^{ks ( \cdot )}\sum _{j = 0}^{k} \bigl\vert {T}_{\sigma_{j,k,\ell}} ({f},{g}) \bigr\vert \Biggr\} _{k} \Biggr\Vert _{\ell^{q( \cdot)}(L^{p( \cdot)})}\\\quad \le C \Vert \sigma \Vert _{N,M}2^{ - \ell N} \Biggl\Vert \Biggl( 2^{ks ( \cdot )}\Psi_{k}^{ * \frac{a}{2}}f\sum _{j = 0}^{\infty} \Psi_{j}^{ * \frac{a}{2}}g \Biggr)_{k} \Biggr\Vert _{\ell^{q( \cdot)}(L^{p( \cdot)})} \\\quad \le C \Vert \sigma \Vert _{N,M}2^{ - \ell N} \bigl\Vert \bigl( 2^{ks ( \cdot )}\Psi_{k}^{ * \frac{a}{2}}f \bigr)_{k} \bigr\Vert _{\ell^{q( \cdot)}(L^{p_{1}( \cdot)})} \Biggl\Vert \sum_{j = 0}^{\infty} \Psi_{j}^{ * \frac{a}{2}}g \Biggr\Vert _{L^{p_{2} ( \cdot )}} \\\quad \le C \Vert \sigma \Vert _{N,M}2^{ - \ell N} \Vert f \Vert _{B_{p_{1}( \cdot),q( \cdot)}^{s( \cdot)}} \Vert g \Vert _{F_{p_{2}( \cdot),1}^{0}}, \end{gathered} $$

where we used Lemma 2.17 and Lemma 2.4 by choosing ${a} > \frac{{n}+ {C}_{\log} ( {1} / {{q}} )}{ \min ( {p}_{1}^{-}, {p}_{2}^{-} )} + {C}_{\log} ( {s} )$ such that $M-n > a$ since ${M}-{n} > \frac{{n}+ {C}_{\log} ( {1} / {{q}} )}{\min ( {p}_{1}^{-}, {p}_{2}^{-} )} + {C}_{\log} ( {s} )$ by the hypothesis of (b). □

### Lemma 3.6

*Let* ${p}, {p}_{1}, {p}_{2}, q \in \mathcal{P}_{0}^{\log} ( \mathbb{{R}}^{{n}} )$ *such that* $1< p^{-},p_{1}^{-},p_{2}^{-}$, *and* ${p}_{1}^{+}, {p}_{2}^{+}, {p}^{+} ,q^{+}< \infty$ *and* $\frac{1}{{p}({x})} = \frac{1}{ {p}_{1({x})}} + \frac{1}{{p}_{2({x})}}$. *Let* ${N}, {M}\in \mathbb{{N}}_{0}$ *be even numbers*.

- *If* ${s} \in {C}_{{\mathrm{loc}}}^{\log} \cap {L}^{\infty}$ *with* ${s}^{-} > 0$, ${s}^{+} < \infty$, ${M} > \frac{{n}}{\min ( {p}_{1}^{-}, {p}_{2}^{-}, {q}^{-} )} + {C}_{\log} ( {s} ) +{n}$, *then there exists a constant* *C* *depending only on* *N*, *M*, *n* *and* ${p}_{1}$, ${p}_{2}$, *q*, *s* *such that*
  $$\bigg\| \sum_{j,k \in\mathbb{N}_{0},j \le k} {T}_{\sigma_{j,k,\ell}} ({f},{g}) \bigg\| _{F_{p( \cdot),q( \cdot)}^{s( \cdot)}} \le C \Vert \sigma \Vert _{N,M}2^{ ( s^{ +} - N )\ell} \Vert f \Vert _{F_{p_{1}( \cdot),q( \cdot)}^{s( \cdot)}} \Vert g \Vert _{F_{p_{2}( \cdot),1}^{0}} $$
  *for all* $\ell\in\mathbb{{N}}_{0}$, ${f}, {g}\in\mathcal{S} ( \mathbb{{R}}^{{n}} )$ *and* $\sigma\in{B} \mathcal{S}_{1,1}^{0}$.
- *If* ${s} \in {C}_{{\mathrm{loc}}}^{\log} \cap {L}^{\infty}$ *with* ${s}^{-} > 0$, ${s}^{+} < \infty$ *and* ${N} > \frac{{n}+ {C}_{\log} ( {1} / {{q}} )}{\min ( {p}_{1}^{-}, {p}_{2}^{-} )} + {C}_{\log} ( {s} ) +{n}$, *then there exists a constant* *C* *depending only on* *N*, *M*, *n* *and* ${p}_{1}$, ${p}_{2}$, *q*, *s* *such that*
  $$\bigg\| \sum_{j,k \in\mathbb{N}_{0},j \le k} {T}_{\sigma_{j,k,\ell}} ({f},{g}) \bigg\| _{B_{p( \cdot),q( \cdot)}^{s( \cdot)}} \le C \Vert \sigma \Vert _{N,M}2^{ ( s^{ +} - N )\ell} \Vert f \Vert _{B_{p_{1}( \cdot),q( \cdot)}^{s( \cdot)}} \Vert g \Vert _{F_{p_{2}( \cdot),1}^{0}} $$
  *for all* $\ell\in\mathbb{{N}}_{0}, {f}, {g}\in\mathcal{S} ( \mathbb{{R}}^{{n}} )$ *and* $\sigma\in{B} \mathcal{S}_{1,1}^{0}$.

### Proof

Let ${f}, {g}\in\mathcal{S} ( \mathbb{{R}}^{{n}} )$. We shall use the characterizations of Besov and Triebel–Lizorkin spaces with variable exponents by approximation as described in Lemma 2.14 and Theorem 2.15, which require the condition ${s} \in {C}_{{\mathrm{loc}}}^{\log} \cap{L}^{\infty}$. For each fixed $\ell\in \mathbb{{N}}_{0}$, we put

$$h_{\ell}: = \sum_{j,k \in\mathbb{N}_{0},j \le k} T_{\sigma_{j,k,\ell}} ( f,g ). $$

It is enough to estimate $\Vert {h}_{\ell} \Vert _{{F}_{{p} ( {\cdot} ),{q} ( {\cdot} )}^{{s} ( {\cdot} )}}^{\omega_{\ell}}$ and $\Vert {h}_{\ell} \Vert _{{B}_{{p} ( {\cdot} ),{q} ( {\cdot} )}^{{s} ( {\cdot} )}}^{\omega_{\ell}}$ (see (3) and (4)) for an appropriate sequence of functions $\omega_{\ell}$. To do so, we define the sequence $\omega_{\ell} := \{ \omega_{{k}, \ell} \}_{{k}\in\mathbb{{N}}_{0}}$ as follows:

$$\omega_{k,\ell}: = \left \{ \textstyle\begin{array}{l@{\quad}l} 0 & \mbox{if } k \le\ell- 1, \\ \sum_{\nu= 0}^{k - \ell} \sum_{j = 0}^{\nu} T_{\sigma_{j,k,\ell}} ( f, g) & \mbox{if } k \ge\ell. \end{array}\displaystyle \right . $$

Then we have

$$\omega_{k,\ell} \in L^{p( \cdot)} \bigl( \mathbb{R}^{n} \bigr) \cap\mathcal{{S}}' \bigl( \mathbb{R}^{n} \bigr) \quad\mbox{and} \quad\lim_{k \to\infty} \omega_{k,\ell} = h_{\ell} \quad\mbox{in } \mathcal{{S}}' \bigl( \mathbb{R}^{n} \bigr) . $$

We claim that

$$\operatorname{supp} ( \widehat{\omega}_{k,\ell} ) \subset \bigl\{ \zeta\in \mathbb{R}^{n}: \vert \zeta \vert \le2^{k + 3} \bigr\} ,\quad k, \ell\in \mathbb{N}_{0}. $$

This inclusion is induced by the fact that

$$\operatorname{supp} \bigl( \widehat{ T_{\sigma_{j,k,\ell}} ( f, g)} \bigr) \subset \bigl\{ \zeta \in\mathbb{R}^{n}: \vert \zeta \vert \le2^{\nu+ \ell+ 3} \bigr\} \quad\mbox{for all } j,\nu,\ell\in\mathbb{N}_{0},j \le\nu, $$

which is easy to check; see [37].

For ${s}^{-} > 0$, by the generalized Hölder inequality, we have

17 $$ \begin{aligned}[b] \Vert h_{\ell} \Vert _{L^{p( \cdot)}} &= \bigg\| \sum_{j,k \in\mathbb{N}_{0},j \le k} {T}_{\sigma_{j,k,\ell}} ({f},{g}) \bigg\| _{L^{p( \cdot)}} \\ &\le C \Biggl\Vert \Biggl( \sum_{k \in\mathbb{N}_{0}} 2^{ks^{ -} q ( \cdot )} \Biggl( \sum_{j = 0}^{k} \bigl\vert {T}_{\sigma_{j,k,\ell}} ({f},{g}) \bigr\vert \Biggr)^{q ( \cdot )} \Biggr)^{\frac{1}{q ( \cdot )}} \Biggr\Vert _{L^{p( \cdot)}} \\ &\le C \Biggl\Vert \Biggl( \sum_{k \in\mathbb{N}_{0}} 2^{ks ( \cdot )q ( \cdot )} \Biggl( \sum_{j = 0}^{k} \bigl\vert {T}_{\sigma_{j,k,\ell}} ({f},{g}) \bigr\vert \Biggr)^{q ( \cdot )} \Biggr)^{\frac{1}{q ( \cdot )}} \Biggr\Vert _{L^{p( \cdot)}} \\ &\le C \Vert \sigma \Vert _{N,M}2^{ - \ell N} \Vert f \Vert _{F_{p_{1}( \cdot),q( \cdot)}^{s( \cdot)}} \Vert g \Vert _{F_{p_{2}( \cdot),1}^{0}}, \end{aligned} $$

where in the last inequality we used Lemma 3.5. Similarly, we obtain

18 $$ \begin{aligned}[b] \Vert h_{\ell} \Vert _{L^{p( \cdot)}}& = \bigg\| \sum_{j,k \in\mathbb{N}_{0},j \le k} {T}_{\sigma_{j,k,\ell}} ({f},{g}) \bigg\| _{L^{p( \cdot)}} \\ &\le C\bigg\| \sum_{j,k \in\mathbb{N}_{0},j \le k} {T}_{\sigma_{j,k,\ell}} ({f},{g}) \bigg\| _{B_{p( \cdot),q( \cdot)}^{s( \cdot)}} \\ &\le C \Biggl\Vert \Biggl( 2^{ks ( \cdot )}\sum_{j = 0}^{k} {T}_{\sigma_{j,k,\ell}} ({f},{g}) \Biggr)_{k} \Biggr\Vert _{\ell^{q( \cdot)}(L^{p( \cdot)})} \\ &\le C \Vert \sigma \Vert _{N,M}2^{ - \ell N} \Vert f \Vert _{B_{p_{1}( \cdot),q( \cdot)}^{s( \cdot)}} \Vert g \Vert _{F_{p_{2}( \cdot),1}^{0}}, \end{aligned} $$

where the first inequality follows from ${B}_{{p}({\cdot}),q({\cdot})}^{{s}( {\cdot})} \hookrightarrow{L}^{{p}(\cdot)}$ if ${s}^{-} > 0$ by Lemma 2.12 and Remark 2.13, and the last inequality follows from Lemma 3.5.

Notice that the $\omega_{0, \ell} =0$ if $\ell \in\mathbb{{N}}$, $\omega_{0,0} = {T}_{\sigma_{0,0,0}} ({f},{g})$ and that (17) implies that

$$\bigl\Vert T_{\sigma_{0,0,0}}(f,g) \bigr\Vert _{L^{p( \cdot)}} \le C \Vert \sigma \Vert _{N,M} \Vert f \Vert _{F_{p_{1}( \cdot),q( \cdot)}^{s( \cdot)}} \Vert g \Vert _{F_{p_{2}( \cdot),1}^{0}}. $$

Thus we have

$$\Vert \omega_{0,\ell} \Vert _{L^{p( \cdot)}} \le C \Vert \sigma \Vert _{N,M}2^{ - \ell N} \Vert f \Vert _{F_{p_{1}( \cdot),q( \cdot )}^{s( \cdot)}} \Vert g \Vert _{F_{p_{2}( \cdot),1}^{0}} $$

for all $\ell\in \mathbb{{N}}_{0}$. Using (18), we have

$$\Vert \omega_{0,\ell} \Vert _{L^{p( \cdot)}} \le C \Vert \sigma \Vert _{N,M}2^{ - \ell N} \Vert f \Vert _{B_{p_{1}( \cdot),q( \cdot )}^{s( \cdot)}} \Vert g \Vert _{F_{p_{2}( \cdot),1}^{0}} $$

for all $\ell\in \mathbb{{N}}_{0}$. We now estimate $\Vert \{ 2^{{ks}({\cdot})} | {h}_{\ell} - \omega_{{k}, \ell} | \}_{{k} \in\mathbb{{N}}_{0}} \Vert _{{L}^{{p}({\cdot})} ( \ell^{{q}({\cdot})} )}$ by breaking the sum in *k* into ${k}\leq\ell- 1$ and ${k}\geq \ell$. Since $\omega_{{k}, \ell} =0$ if ${k}\leq\ell- 1$, for the first part we obtain

$$\begin{aligned} \Biggl\Vert \Biggl( \sum _{k = 0}^{\ell- 1} \bigl( 2^{ks ( \cdot )} \vert h_{\ell} \vert \bigr)^{q( \cdot)} \Biggr)^{\frac{1}{q( \cdot)}} \Biggr\Vert _{L^{p( \cdot)}} &\le C \Biggl\Vert \Biggl( \sum _{k = 0}^{\ell- 1} \bigl( 2^{ - \vert k - \ell \vert s ( \cdot )}2^{\ell s^{ +}} \vert h_{\ell} \vert \bigr)^{q( \cdot)} \Biggr)^{\frac{1}{q( \cdot)}} \Biggr\Vert _{L^{p( \cdot)}} \\ &\le C2^{\ell s^{ +}} \Vert h_{\ell} \Vert _{L^{p( \cdot)}} \le C \Vert \sigma \Vert _{N,M}2^{\ell ( s^{ +} - N )} \Vert f \Vert _{F_{p_{1}( \cdot),q( \cdot)}^{s( \cdot)}} \Vert g \Vert _{F_{p_{2}( \cdot),1}^{0}}, \end{aligned} $$

where the second inequality follows from Lemma 2.10 and the last inequality follows from (17). Now, we turn to an estimate of the second part (that is, when ${k}\geq\ell$). Since

$$h_{\ell} - \omega_{k,\ell} = \sum_{\nu= k - \ell+ 1}^{\infty} \sum_{j = 0}^{\nu} T_{\sigma_{j,\nu,\ell}} (f,g) = \sum_{\nu= 1}^{\infty} \sum _{j = 0}^{k - \ell+ \nu} T_{\sigma_{j,k - \ell+ \nu,\ell}} (f,g), $$

we have

$$\begin{gathered} \bigl\Vert \bigl\{ 2^{ks ( \cdot )} \vert h_{\ell} - \omega_{k,\ell} \vert \bigr\} _{k = \ell}^{\infty} \bigr\Vert _{L^{p( \cdot)}(\ell^{q( \cdot)})} \\\quad\le \Biggl\Vert \Biggl\{ 2^{ks ( \cdot )}\sum _{\nu= 1}^{\infty} \sum _{j = 0}^{k - \ell+ \nu} T_{\sigma_{j,k - \ell+ \nu,\ell}} (f,g) \Biggr\} _{k = \ell}^{\infty} \Biggr\Vert _{L^{p( \cdot)}(\ell^{q( \cdot)})} \\ \quad= \Biggl\Vert \Biggl\{ \sum_{\nu= 1}^{\infty} 2^{ ( \ell- \nu )s ( \cdot )}2^{ ( k - \ell+ \nu )s ( \cdot )}\sum_{j = 0}^{k - \ell+ \nu} \bigl\vert T_{\sigma_{j,k - \ell+ \nu,\ell}} (f,g) \bigr\vert \Biggr\} _{k = \ell}^{\infty} \Biggr\Vert _{L^{p( \cdot )}(\ell^{q( \cdot)})} \\ \quad\le\sum_{\nu= 1}^{\infty} 2^{ ( \ell- \nu )s ( \cdot )} \Biggl\Vert \Biggl\{ 2^{ ( k - \ell+ \nu )s ( \cdot )}\sum_{j = 0}^{k - \ell+ \nu} \bigl\vert T_{\sigma_{j,k - \ell+ \nu,\ell}} (f,g) \bigr\vert \Biggr\} _{k = \ell}^{\infty} \Biggr\Vert _{L^{p( \cdot )}(\ell^{q( \cdot)})} \\ \quad\le C\sum_{\nu= 1}^{\infty} 2^{ ( \ell- \nu )s ( \cdot )} \Vert \sigma \Vert _{N,M}2^{ - \ell N} \Vert f \Vert _{F_{p_{1}( \cdot),q( \cdot)}^{s( \cdot)}} \Vert g \Vert _{F_{p_{2}( \cdot),1}^{0}} \\ \quad\le C\sum_{\nu= 1}^{\infty} 2^{ ( \ell- \nu )s^{ +}} \Vert \sigma \Vert _{N,M}2^{ - \ell N} \Vert f \Vert _{F_{p_{1}( \cdot),q( \cdot)}^{s( \cdot)}} \Vert g \Vert _{F_{p_{2}( \cdot),1}^{0}} \\ \quad\le C \Vert \sigma \Vert _{N,M}2^{\ell ( s^{ +} - N )} \Vert f \Vert _{F_{p_{1}( \cdot),q( \cdot)}^{s( \cdot)}} \Vert g \Vert _{F_{p_{2}( \cdot),1}^{0}}, \end{gathered} $$

where the third inequality follows from Lemma 3.5.

Then we estimate

$$\bigl\Vert \bigl\{ 2^{ks ( \cdot )} \vert h_{\ell} - \omega _{k,\ell} \vert \bigr\} _{k = \ell}^{\infty} \bigr\Vert _{\ell^{q( \cdot)}(L^{p( \cdot)})} \le C \Vert \sigma \Vert _{N,M}2^{\ell ( s^{ +} - N )} \Vert f \Vert _{B_{p_{1}( \cdot),q( \cdot)}^{s( \cdot )}} \Vert g \Vert _{F_{p_{2}( \cdot),1}^{0}}. $$

Since $\omega_{{k}, \ell} =0$ if ${k}\leq \ell- 1$, for the first part we obtain

$$\sum_{k = 0}^{\ell- 1} 2^{ks ( \cdot )} h_{\ell} = \sum_{k = 0}^{\ell- 1} 2^{ - \vert k - \ell \vert s ( \cdot )} 2^{\ell s ( \cdot )}h_{\ell} \le2^{\ell s^{ +}} \sum _{k = 0}^{\ell- 1} 2^{ - \vert k - \ell \vert s ( \cdot )} h_{\ell}. $$

Then by Lemma 2.10, we have

$$\begin{aligned} \Biggl\Vert \sum_{k = 0}^{\ell- 1} 2^{ks ( \cdot )} h_{\ell} \Biggr\Vert _{L^{p( \cdot)}} &\le2^{\ell s^{ +}} \Biggl\Vert \sum_{k = 0}^{\ell- 1} 2^{ - \vert k - \ell \vert s ( \cdot )} h_{\ell} \Biggr\Vert _{L^{p( \cdot)}} \\ &\le C2^{\ell s^{ +}} \Vert h_{\ell} \Vert _{L^{p( \cdot)}} \\ &\le C \Vert \sigma \Vert _{N,M}2^{\ell ( s^{ +} - N )} \Vert f \Vert _{B_{p_{1}( \cdot),q( \cdot)}^{s( \cdot)}} \Vert g \Vert _{F_{p_{2}( \cdot),1}^{0}}, \end{aligned} $$

where the last inequality follows from (18). Since

$$\begin{aligned} \bigl( 2^{ks ( \cdot )} ( h_{\ell} - \omega_{k,\ell} ) \bigr)_{{k}}& = \Biggl( 2^{ks ( \cdot )}\sum _{\nu= 1}^{\infty} \sum _{j = 0}^{k - \ell+ \nu} T_{\sigma_{j,k - \ell+ \nu,\ell}} (f,g) \Biggr)_{{k}} \\ &= \Biggl( 2^{ ( \ell- \nu )s ( \cdot )}2^{ ( k - \ell+ \nu )s ( \cdot )}\sum_{\nu= 1}^{\infty} \sum_{j = 0}^{k - \ell+ \nu} T_{\sigma_{j,k - \ell+ \nu,\ell}} (f,g) \Biggr)_{{k}} \\ &= \sum_{\nu= 1}^{\infty} 2^{ ( \ell- \nu )s ( \cdot )} \Biggl( 2^{ ( k - \ell+ \nu )s ( \cdot )}\sum_{j = 0}^{k - \ell+ \nu} T_{\sigma_{j,k - \ell+ \nu,\ell}} (f,g) \Biggr)_{{k}}. \end{aligned} $$

Let ${r}\in ( 0, \frac{1}{2} \min \{ {p}^{-}, {q}^{-},2 \} )$, by using Lemma 2.11, we obtain

$$\begin{gathered} \bigl\Vert \bigl( 2^{ks ( \cdot )} ( h_{\ell } - \omega_{k,\ell} ) \bigr)_{{k}} \bigr\Vert _{\ell^{q( \cdot )}(L^{p( \cdot)})}\\\quad = \Biggl\Vert \sum_{\nu= 1}^{\infty} 2^{ ( \ell- \nu )s ( \cdot )} \Biggl( 2^{ ( k - \ell+ \nu )s ( \cdot )}\sum_{j = 0}^{k - \ell+ \nu} T_{\sigma _{j,k - \ell+ \nu,\ell}} (f,g) \Biggr)_{{k}} \Biggr\Vert _{\ell^{q( \cdot )} ( L^{p( \cdot)} )} \\ \quad= \Bigg\| \Bigg| \sum_{\nu= 1}^{\infty} 2^{ ( \ell- \nu )s ( \cdot )} \Biggl( 2^{ ( k - \ell+ \nu )s ( \cdot )}\sum_{j = 0}^{k - \ell+ \nu} T_{\sigma _{j,k - \ell+ \nu,\ell}} (f,g) \Biggr)_{{k}}\Bigg|^{r} \Bigg\| ^{\frac{1}{r}}_{\ell^{\frac{q( \cdot)}{r}}(L^{\frac{p( \cdot )}{r}})} \\ \quad\le \Biggl\Vert \sum_{\nu= 1}^{\infty} 2^{ ( \ell- \nu )rs ( \cdot )} \Biggl( 2^{ ( k - \ell+ \nu )rs ( \cdot )} \Biggl\vert \sum _{j = 0}^{k - \ell+ \nu} T_{\sigma_{j,k - \ell+ \nu,\ell}} (f,g) \Biggr\vert ^{r} \Biggr)_{{k}} \Biggr\Vert ^{\frac{1}{r}}_{\ell^{\frac{q( \cdot)}{r}}(L^{\frac{p( \cdot )}{r}})} \\ \quad\le \Biggl( \sum_{\nu= 1}^{\infty} 2^{ ( \ell- \nu )rs^{ +}} \Biggl\Vert \Biggl( 2^{ ( k - \ell+ \nu )rs ( \cdot )} \Biggl\vert \sum _{j = 0}^{k - \ell+ \nu} T_{\sigma_{j,k - \ell+ \nu,\ell}} (f,g) \Biggr\vert ^{r} \Biggr)_{{k}} \Biggr\Vert _{\ell^{\frac{q( \cdot)}{r}}(L^{\frac{p( \cdot)}{r}})} \Biggr)^{\frac{1}{r}} \\ \quad\le C\sum_{\nu= 1}^{\infty} 2^{ ( \ell- \nu )s^{ +}} \Biggl\Vert \Biggl( 2^{ks ( \cdot )}\sum_{j = 0}^{k} \bigl\vert T_{\sigma_{j,k,\ell}} (f,g) \bigr\vert \Biggr)_{k} \Biggr\Vert _{\ell^{q( \cdot )}(L^{p( \cdot)})} \\ \quad\le C2^{\ell ( s^{ +} - N )} \Vert \sigma \Vert _{N,M} \Vert f \Vert _{B_{p_{1}( \cdot),q( \cdot)}^{s( \cdot)}} \Vert g \Vert _{F_{p_{2}( \cdot),1}^{0}}, \end{gathered} $$

where in the last inequality we used Lemma 3.5. Thus, we obtain

$$\begin{aligned} \Vert h_{\ell} \Vert _{F_{p( \cdot),q( \cdot)}^{s( \cdot)}} &\le C \Vert \omega_{0,\ell} \Vert _{L^{p( \cdot)}} + \bigl\Vert \bigl\{ 2^{ks ( \cdot )} \vert h_{\ell} - \omega_{k,\ell} \vert \bigr\} _{k \in\mathbb{N}_{0}} \bigr\Vert _{L^{p( \cdot)}(\ell^{q( \cdot )})} \\ &\le C \Vert \sigma \Vert _{N,M}2^{\ell ( s^{ +} - N )} \Vert f \Vert _{F_{p_{1}( \cdot),q( \cdot)}^{s( \cdot)}} \Vert g \Vert _{F_{p_{2}( \cdot),1}^{0}} \end{aligned} $$

and

$$\begin{aligned} \Vert h_{\ell} \Vert _{B_{p( \cdot),q( \cdot)}^{s( \cdot)}} &\le C \Vert \omega_{0,\ell} \Vert _{L^{p( \cdot)}} + \bigl\Vert \bigl\{ 2^{ks ( \cdot )} \vert h_{\ell} - \omega_{k,\ell} \vert \bigr\} _{k \in\mathbb{N}_{0}} \bigr\Vert _{\ell^{q( \cdot)}(L^{p( \cdot )})} \\ &\le C \Vert \sigma \Vert _{N,M}2^{\ell ( s^{ +} - N )} \Vert f \Vert _{B_{p_{1}( \cdot),q( \cdot)}^{s( \cdot)}} \Vert g \Vert _{F_{p_{2}( \cdot),1}^{0}}, \end{aligned} $$

as desired. □

After these preparation, we now complete the proofs of Theorems 3.1 and 3.2.

### Proofs of Theorems 3.1 and 3.2

We firstly conclude the proofs of (8) and (10). Since

$$I_{1} ( x ) = \sum_{\ell\in\mathbb{N}_{0}}^{\infty} \sum_{j,k \in\mathbb{N}_{0},j \le k} T_{\sigma_{j,k,\ell}} (f,g) ( x ), $$

by choosing ${N} > {s}^{+}$, part (a) of Lemma 3.6, we obtain

$$\Vert I_{1} \Vert _{F_{p( \cdot),q( \cdot)}^{s( \cdot)}} \le C \Vert \sigma \Vert _{N,M} \Vert f \Vert _{F_{p_{1}( \cdot),q( \cdot)}^{s( \cdot)}} \Vert g \Vert _{F_{p_{2}( \cdot),1}^{0}}. $$

By interchanging the roles of *j* and *k*, *f* and *g*, *ξ* and *η*, we have

$$\Vert I_{2} \Vert _{F_{p( \cdot),q( \cdot)}^{s( \cdot)}} \le C \Vert \sigma \Vert _{N,M} \Vert f \Vert _{F_{p_{1}( \cdot ),1}^{0}} \Vert g \Vert _{F_{p_{2}( \cdot),q( \cdot)}^{s( \cdot)}}. $$

Hence the proof of (8) is complete. Similarly, we obtain the inequality (10) by using part (b) of Lemma 3.6.

By Lemma 2.12, the inequalities (9) and (11) follow from (8) and (10), respectively, since

$$\begin{gathered} {F}_{{p}_{2} ({\cdot}),q({\cdot})}^{{s}({\cdot})} \hookrightarrow{B}_{{p}_{2} ({\cdot}),\max \{ {p}_{2} ({\cdot}),q({\cdot}) \}}^{{s}({\cdot})} \hookrightarrow {B}_{{p}_{2} ({\cdot}),\min \{ {p}_{2} ({\cdot}),q({\cdot}) \}}^{0} \hookrightarrow {F}_{{p}_{2} ( {\cdot} ),1}^{0}, \\ {F}_{{p}_{1} ({\cdot}),q({\cdot})}^{{s}({\cdot})} \hookrightarrow{B}_{{p}_{1} ({\cdot}),\max \{ {p}_{2} ({\cdot}),q({\cdot}) \}}^{{s}({\cdot})} \hookrightarrow {B}_{{p}_{1} ({\cdot}),\min \{ {p}_{2} ({\cdot}),q({\cdot}) \}}^{0} \hookrightarrow {F}_{{p}_{1} ({\cdot}),1}^{0},\end{gathered} $$

and

$$\begin{gathered} {B}_{{p}_{2} ({\cdot}),q({\cdot})}^{{s}({\cdot})} \hookrightarrow{B}_{{p}_{2} ({\cdot}),\min \{ {p}_{2} ({\cdot}),1 \}}^{0} \hookrightarrow {F}_{{p}_{2} ( {\cdot} ),1}^{0}, \\ {B}_{{p}_{1} ({\cdot}),q({\cdot})}^{{s}({\cdot})} \hookrightarrow{B}_{{p}_{1} ({\cdot}),\min \{ {p}_{1} ({\cdot}),1 \}}^{0} \hookrightarrow {F}_{{p}_{1} ({\cdot}),1}^{0}.\end{gathered} $$

Thus the proof finishes. □

## References

[1] Almeida A., Drihem D. (2012). Maximal, potential and singular type operators on Herz spaces with variable exponts. J. Math. Anal. Appl..

[2] Almeida A., Hasanov J., Samko S. (2008). Maximal and potential operators in variable exponent Morrey spaces. Georgian Math. J..

[3] Almeida A., Hästö P. (2010). Besov spaces with variable smoothness and integrability. J. Funct. Anal..

[4] Almeida A., Samko S. (2006). Characterization of Riesz and Bessel potentials on variable Lebesgue spaces. J. Funct. Spaces Appl..

[5] Almeida A., Samko S. (2009). Embeddings of variable Hajłasz–Sobolev spaces into Hölder spaces of variable order. J. Math. Anal. Appl..

[6] Bényì Á., Bernicot F., Maldonado D., Naibo V., Torres R.H. (2013). On the Hörmander classes of bilinear pseudodifferential operators II. Indiana Univ. Math. J..

[7] Bényì Á., Maldonado D., Naibo V., Torres R.H. (2010). On the Hörmander classes of bilinear pseu dodifferential operators. Integral Equ. Oper. Theory.

[8] Bényì Á., Torres R.H. (2003). Symbolic calculus and the transposes of bilinear psedodifferential operators. Commun. Partial Differ. Equ..

[9] Chen Y., Levine S., Rao R. (2006). Variable exponent, linear growth functionals in image restoration. SIAM J. Appl. Math..

[10] Cruz-Uribe D., Fiorenza A., Neugebauer C. (2003). The maximal function on variable ${L}^{{p}}$ spaces. Ann. Acad. Sci. Fenn., Math..

[11] Diening L. (2004). Maximal function on generalized Lebesgue spaces $L^{p(\cdot)}$. Math. Inequal. Appl..

[12] Diening L., Harjulehto P., Hästö P., Růžička M. (2011). Lebesgue and Sobolev Spaces with Variable Exponents.

[13] Diening L., Hästö P., Roudenko S. (2009). Function spaces of variable smoothness and integrability. J. Funct. Anal..

[14] Dong B., Xu J. (2012). New Herz type Besov and Triebel–Lizorkin spaces with variable exponents. J. Funct. Spaces Appl..

[15] Dong B., Xu J. (2015). Herz–Morrey type Besov and Triebel–Lizorkin spaces with variable exponents. Banach J. Math. Anal..

[16] Drihem D. (2012). Atomic decomposition of Besov spaces with variable smoothness and integrability. J. Math. Anal. Appl..

[17] Drihem D., Heraiz R. (2017). Herz-type Besov spaces of variable smoothness and integrability. Kodai Math. J..

[18] Drihem D., Seghiri F. (2016). Notes on the Herz-type Hardy spaces of variable smoothness and integrability. Math. Inequal. Appl..

[19] Edmunds D.E., Lang J., Mendez O. (2014). Differential Operators on Spaces of Variable Integrability.

[20] Fu J., Xu J. (2011). Characterizations of Morrey type Besov and Triebel–Lizorkin spaces with variable exponents. J. Math. Anal. Appl..

[21] Gurka P., Harjuleto P., Nekvinda A. (2007). Bessel potential spaces with variable exponent. Math. Inequal. Appl..

[22] Harjulehto P., Hästö P., Latvala V., Toivanen O. (2013). Critical variable exponent functionals in image restoration. Appl. Math. Lett..

[23] Harjulehto P., Hästö P., Le U.V., Nuortio M. (2010). Overview of differential equations with nonstandard growth. Nonlinear Anal..

[24] Herbert J., Naibo V. (2014). Bilinear pseudodifferential operators with symbols in Besov spaces. J. Pseudo-Differ. Oper. Appl..

[25] Huang A., Xu J. (2010). Multilinear singular integrals and commutators in variable exponent Lebesgue spaces. Appl. Math. J. Chin. Univ. Ser. A.

[26] Izuki M. (2010). Boundedness of sublinear operators on Herz spaces with variable exponent and application to wavelet characterization. Anal. Math..

[27] Izuki M., Noi T. (2014). Duality of Besov, Triebel–Lizorkin and Herz spaces with variable exponents. Rend. Circ. Mat. Palermo.

[28] Izuki M., Noi T. (2016). Hardy spaces associated to critical Herz spaces with variable exponent. Mediterr. J. Math..

[29] Jia J., Peng J., Gao J. (2016). Bayesian approach to inverse problems for functions with variable index Besov prior. Inverse Probl..

[30] Kempka H. (2009). 2-Microlocal Besov and Triebel–Lizorkin spaces of variable integrability. Rev. Mat. Complut..

[31] Kempka H., Vybíral J. (2012). Spaces of variable smoothness and integrability: characterizations by local means and ball means of differences. J. Fourier Anal. Appl..

[32] Kokilashvili V., Meskhi A., Rafeiro H., Samko S. (2016). Integral Operators in Non-standard Function Spaces, Volume 1: Variable Exponent Lebesgue and Amalgam Spaces.

[33] Kokilashvili V., Meskhi A., Rafeiro H., Samko S. (2016). Integral Operators in Non-standard Function Spaces, Volume 2: Variable Exponent Hölder, Morrey–Campanato and Grand Spaces.

[34] Li F., Li Z., Pi L. (2010). Variable exponent functionals in image restoration. Appl. Math. Comput..

[35] Michalowski N., Rule D., Staubach W. (2014). Multilinear pseudodifferential operators beyond Calderón–Zygmund theory. J. Math. Anal. Appl..

[36] Miyachi A., Tomita N. (2013). Calderón–Vaillancourt-tyepe theorem for bilinear operators. Indiana Univ. Math. J..

[37] Naibo V. (2015). On the bilinear Hörmander classes in the scales of Triebel–Lizorkin and Besov spaces. J. Fourier Anal. Appl..

[38] Nakai E., Sawano Y. (2012). Hardy spaces with variable exponents and generalized Campanato spaces. J. Funct. Anal..

[39] Noi T. (2014). Fourier multiplier theorems for Besov and Triebel–Lizorkin spaces with variable exponents. Math. Inequal. Appl..

[40] Peetre J. (1975). On spaces of Triebel–Lizorkin type. Ark. Mat..

[41] Rădulescu V.D., Repovš D.D. (2015). Partial Differential Equations with Variable Exponents.

[42] Rodríguez-López S., Staubach W. (2013). Estimates for rough Fourier integral and pseudodifferential operators and applications to the boundedness of multilinear operators. J. Funct. Anal..

[43] Růžička M. (2000). Electrorheological Fluids: Modeling and Mathematical Theory.

[44] Samko S. (2013). Variable exponent Herz spaces. Mediterr. J. Math..

[45] Shi C., Xu J. (2013). Herz type Besov and Triebel–Lizorkin spaces with variable exponent. Front. Math. China.

[46] Tiirola J. (2014). Image decompositions using spaces of variable smoothness and integrability. SIAM J. Imaging Sci..

[47] Triebel H. (1983). Theory of Function Spaces.

[48] Wang H., Liu Z. (2012). The Herz-type Hardy spaces with variable exponent and their applications. Taiwan. J. Math..

[49] Xu J. (2008). Variable Besov spaces and Triebel–Lizorkin spaces. Ann. Acad. Sci. Fenn., Math..

[50] Xu J., Yang X. (2016). The molecular decomposition of Herz–Morrey–Hardy spaces with variable exponents and its application. J. Math. Inequal..

[51] Xu J., Yang X. (2017). Variable integral and smooth exponent Triebel–Lizorkin spaces associated with a non-negative self-adjoint operator. Math. Inequal. Appl..

[52] Xu J., Yang X. (2018). Variable exponent Herz type Besov and Triebel–Lizorkin spaces. Georgian Math. J..

[53] Yan X., Yang D., Yuan W., Zhuo C. (2016). Variable weak Hardy spaces and their applications. J. Funct. Anal..

[54] Yang D., Zhang J., Zhuo C. (2018). Variable Hardy spaces associated with operators satisfying Davies–Gaffney estimates. Proc. Edinb. Math. Soc..

[55] Yang D., Zhuo C. (2016). Molecular characterizations and dualities of variable exponent Hardy spaces associated with operators. Ann. Acad. Sci. Fenn., Math..

[56] Yang D., Zhuo C., Nakai E. (2016). Characterizations of variable exponent Hardy spaces via Riesz transforms. Rev. Mat. Complut..

[57] Yang D., Zhuo C., Yuan W. (2015). Triebel–Lizorkin type spaces with variable exponents. Banach J. Math. Anal..

[58] Yang D., Zhuo C., Yuan W. (2015). Besov-type spaces with variable smoothness and integrability. J. Funct. Anal..

[59] Zhuo C., Yang D. (2016). Maximal function characterizations of variable Hardy spaces associated with non-negative self-adjoint operators satisfying Gaussian estimates. Nonlinear Anal..

[60] Zhuo C., Yang D., Liang Y. (2016). Intrinsic square function characterizations of Hardy spaces with variable exponents. Bull. Malays. Math. Sci. Soc..

